# Supplementary material for: Detection of Adulterated Naodesheng Tablet (Naodesheng Pian) via In-Depth Chemical Analysis and Subsequent Reconstruction of Its Pharmacopoeia Q-Markers
Source: Molecules. 2024 Mar 20;29(6):1392. doi: 10.3390/molecules29061392 (PMC10974483; doi:10.3390/molecules29061392)
Supplement: Supplementary file 1 [file molecules-29-01392-s001.zip › molecules-2869443-supplementary.pdf]

Suppl. 1 Identification of D-gluconic acid CAS 526-95-41

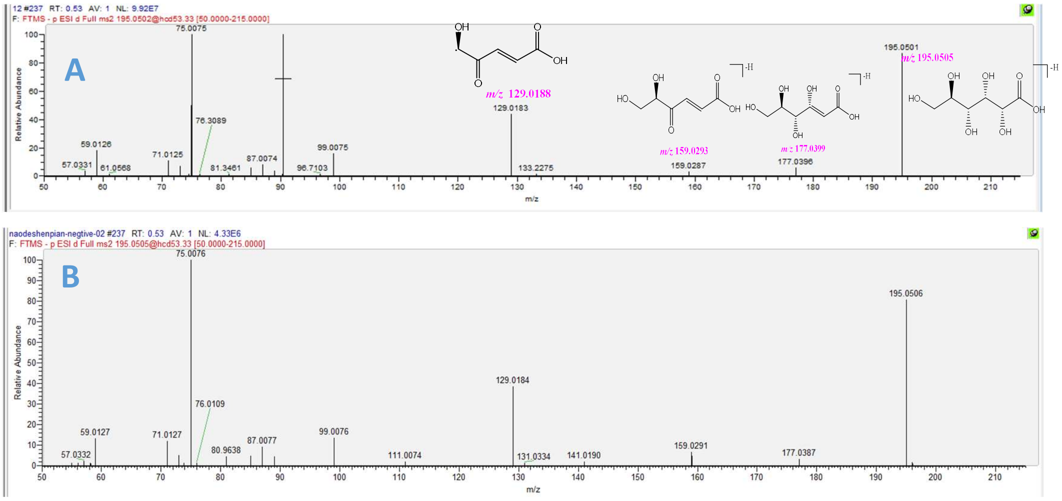

Fig. S1.1 The main results of standard D-gluconic acid (CAS 526-95-4, $C_6H_{12}O_7$) and its corresponding peak in the TIC diagram using UPLC-Q-Orbitrap-MS analysis. (**A**) The MS/MS fragments of standard D-gluconic acid. (**B**) The MS/MS spectra from chromatographic peak in the Naodeshengpian extract.

Note: The *m/z* values in purple are the calculated ones. The *m/z* calculation was based on the relative atomic masses of C (12.0000), H (1.007825), O (15.994915), and N (14.003074)[1].

Identification: As seen in Fig. S1.1, the R.T. value, molecular ion peak, MS/MS spectra, and characteristic pears were highly similar. Thus, the chromatographic peak in the Naodeshengpian extract was identified as D-gluconic acid (CAS 526-95-4, $C_6H_{12}O_7$).

Suppl. 2 Identification of citric acid CAS 77-92-9

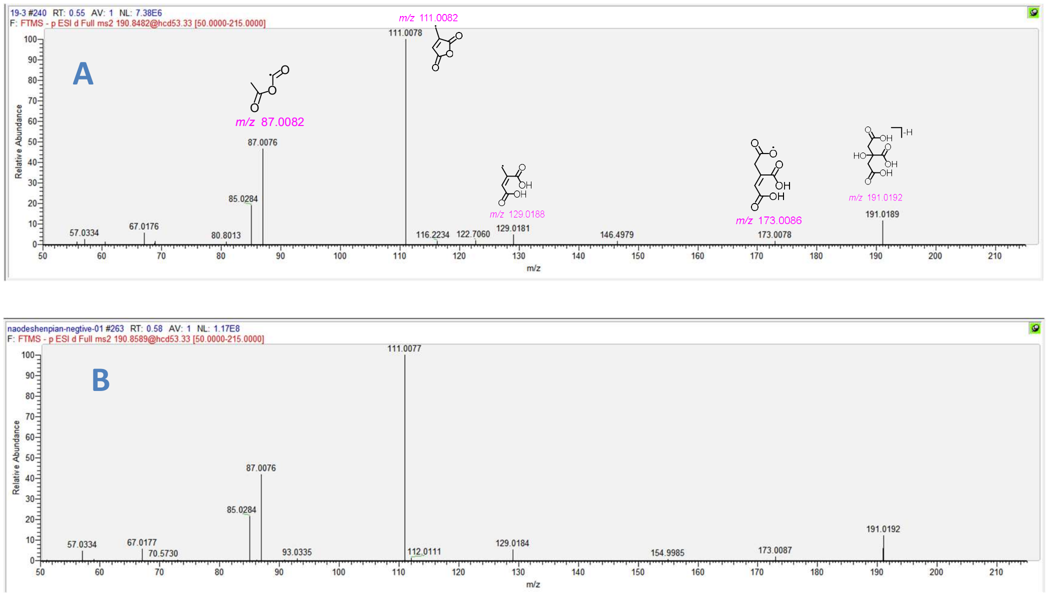

Fig. S2.1 The main results of standard citric acid (CAS 77-92-9, $C_6H_8O_7$)and its corresponding peak in the TIC diagram using UPLC-Q-Orbitrap-MS analysis. (**A**) The MS/MS fragments of standard citric acid. (**B**) The MS/MS spectra from chromatographic peak in the Naodeshengpian extract.

Note: The *m/z* values in purple are the calculated ones. The *m/z* calculation was based on the relative atomic masses of C (12.0000), H (1.007825), O (15.994915), and N (14.003074)[1].

Identification: As seen in Fig. S2.1, the R.T. value, molecular ion peak, MS/MS spectra, and characteristic pears were highly similar. Thus, the chromatographic peak in the Naodeshengpian extract was identified as citric acid (CAS 77-92-9, $C_6H_8O_7$).

Suppl. 3 Identification of phenylalanine CAS 150-30-1

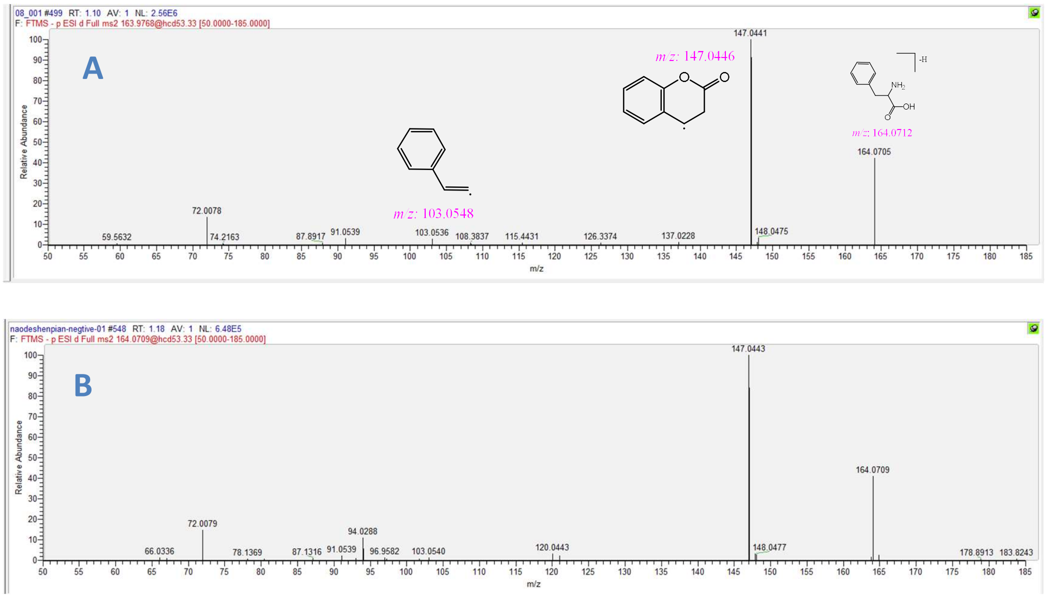

Fig. S3.1 The main results of standard phenylalanine (CAS 150-30-1, $C_9H_{11}NO_2$)and its corresponding peak in the TIC diagram using UPLC-Q-Orbitrap-MS analysis. (**A**) The MS/MS fragments of standard phenylalanine. (**B**) The MS/MS spectra from chromatographic peak in the Naodeshengpian extract.

Note: The *m/z* values in purple are the calculated ones. The *m/z* calculation was based on the relative atomic masses of C (12.0000), H (1.007825), O (15.994915), and N (14.003074)[1].

Identification: As seen in Fig. S3.1, the R.T. value, molecular ion peak, MS/MS spectra, and characteristic pears were highly similar. Thus, the chromatographic peak in the Naodeshengpian extract was identified as phenylalanine(CAS 150-30-1, $C_9H_{11}NO_2$).

Suppl. 4 Identification of protocatechuic acid CAS 99-50-3

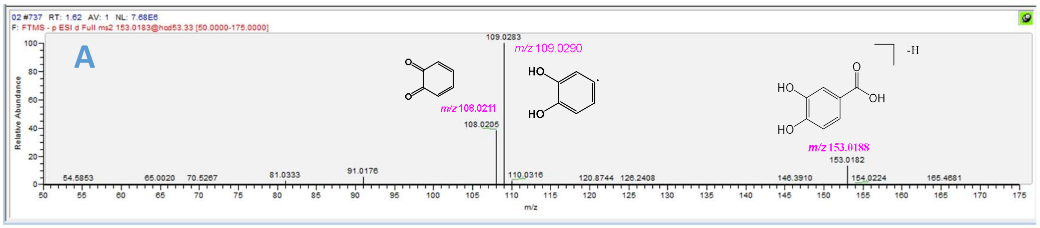

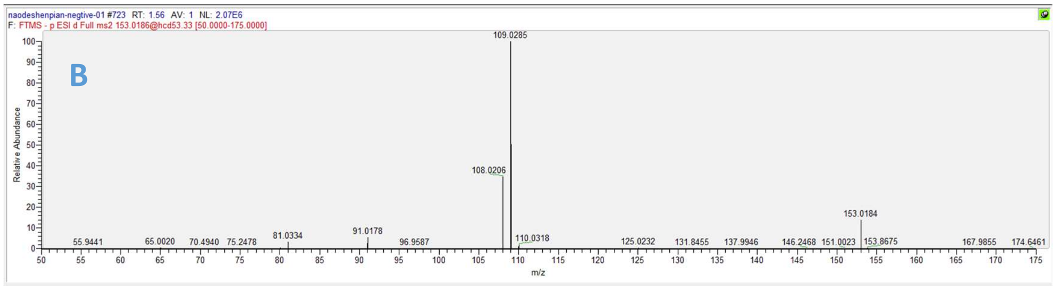

Fig. S4.1 The main results of standard protocatechuate(CAS 99-50-3, $C_7H_6O_4$)and its corresponding peak in the TIC diagram using UPLC-Q-Orbitrap-MS analysis. (**A**) The MS/MS fragments of standard protocatechuate. (**B**) The MS/MS spectra from chromatographic peak in the Naodeshengpian extract.

Note: The *m/z* values in purple are the calculated ones. The *m/z* calculation was based on the relative atomic masses of C (12.0000), H (1.007825), O (15.994915), and N (14.003074)[1].

Identification: As seen in Fig. S4.1, the R.T. value, molecular ion peak, MS/MS spectra, and characteristic pears were highly similar. Thus, the chromatographic peak in the Naodeshengpian extract was identified as protocatechuate(CAS 99-50-3, $C_7H_6O_4$).

Suppl. 5 Identification of L-tryptophan CAS 73-22-3

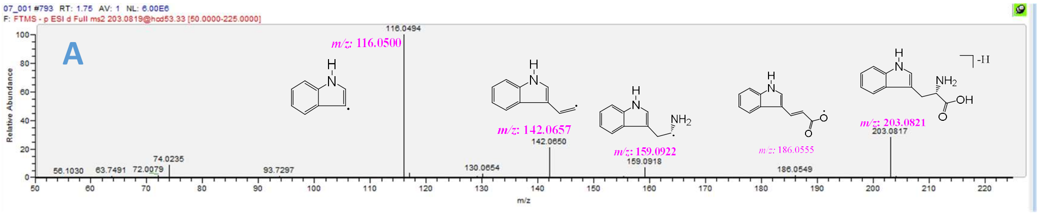

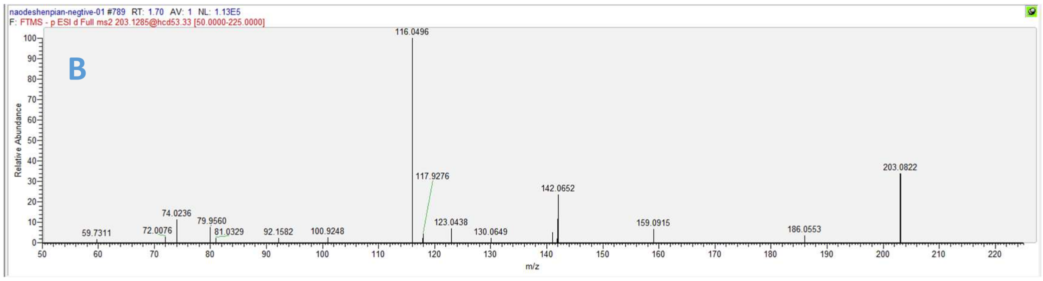

Fig. S5.1 The main results of standard L-tryptophan(CAS 73-22-3, $C_{11}H_{12}N_2O_2$)and its corresponding peak in the TIC diagram using UPLC-Q-Orbitrap-MS analysis. (**A**) The MS/MS fragments of standard L-tryptophan. (**B**) The MS/MS spectra from chromatographic peak in the Naodeshengpian extract.

Note: The *m/z* values in purple are the calculated ones. The *m/z* calculation was based on the relative atomic masses of C (12.0000), H (1.007825), O (15.994915), and N (14.003074)[1].

Identification: As seen in Fig. S5.1, the R.T. value, molecular ion peak, MS/MS spectra, and characteristic pears were highly similar. Thus, the chromatographic peak in the Naodeshengpian extract was identified as L-tryptophan(CAS 73-22-3, $C_{11}H_{12}N_2O_2$).

Suppl. 6 Identification of chlorogenic acid

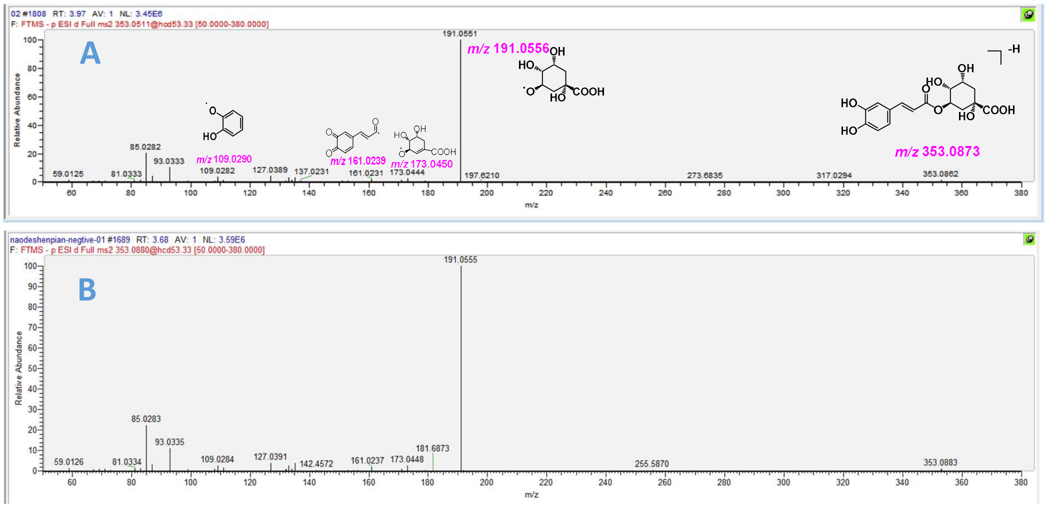

Fig. S6.1 The main results of standard chlorogenic acid (CAS 327-97-9, $C_{16}H_{18}O_9$)and its corresponding peak in the TIC diagram using UPLC-Q-Orbitrap-MS analysis. (**A**) The MS/MS fragments of standard chlorogenic acid. (**B**) The MS/MS spectra from chromatographic peak in the Naodeshengpian extract.

Note: The *m/z* values in purple are the calculated ones. The *m/z* calculation was based on the relative atomic masses of C (12.0000), H (1.007825), O (15.994915), and N (14.003074)[1].

Identification: As seen in Fig. S6.1, the R.T. value, molecular ion peak, MS/MS spectra, and characteristic pears were highly similar. Thus, the chromatographic peak in the Naodeshengpian extract was identified as chlorogenic acid (CAS 327-97-9, $C_{16}H_{18}O_9$).

Suppl. 7 Identification of hydroxy safflor yellow A CAS 78281-02-4

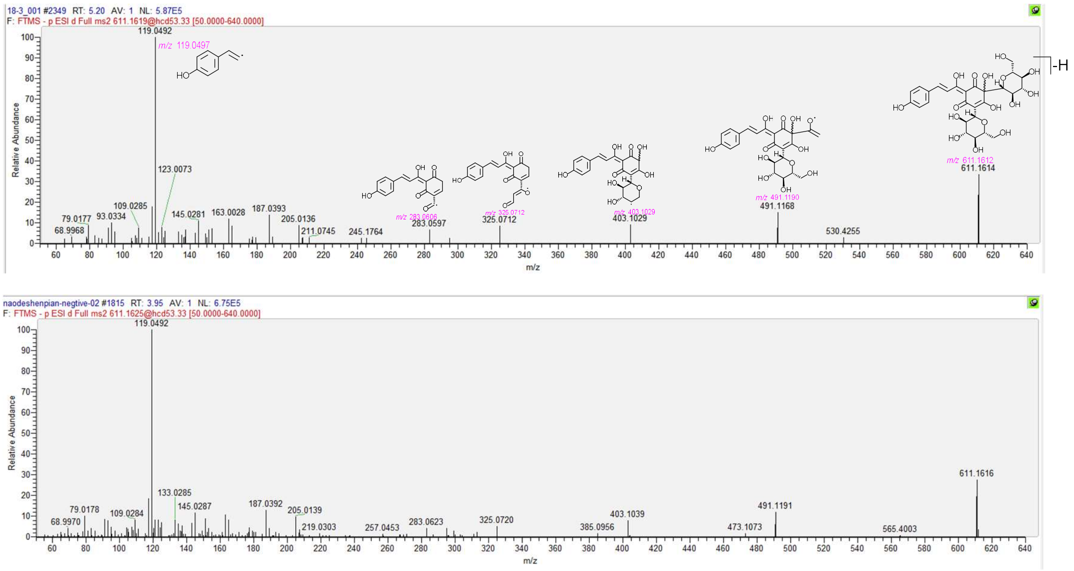

Fig. S7.1 The main results of standard hydroxy safflor yellow A(CAS 78281-02-4, $C_{27}H_{32}O_{16}$)and its corresponding peak in the TIC diagram using UPLC-Q-Orbitrap-MS analysis. (**A**) The MS/MS fragments of standard hydroxy safflor yellow A. (**B**) The MS/MS spectra from chromatographic peak in the Naodeshengpian extract.

Note: The *m/z* values in purple are the calculated ones. The *m/z* calculation was based on the relative atomic masses of C (12.0000), H (1.007825), O (15.994915), and N (14.003074)[1].

Identification: As seen in Fig. S7.1, the R.T. value, molecular ion peak, MS/MS spectra, and characteristic pears were highly similar. Thus, the chromatographic peak in the Naodeshengpian extract was identified as hydroxy safflor yellow A(CAS 78281-02-4, $C_{27}H_{32}O_{16}$).

Suppl. 8 Identification of vanillic acid CAS 121-34-6

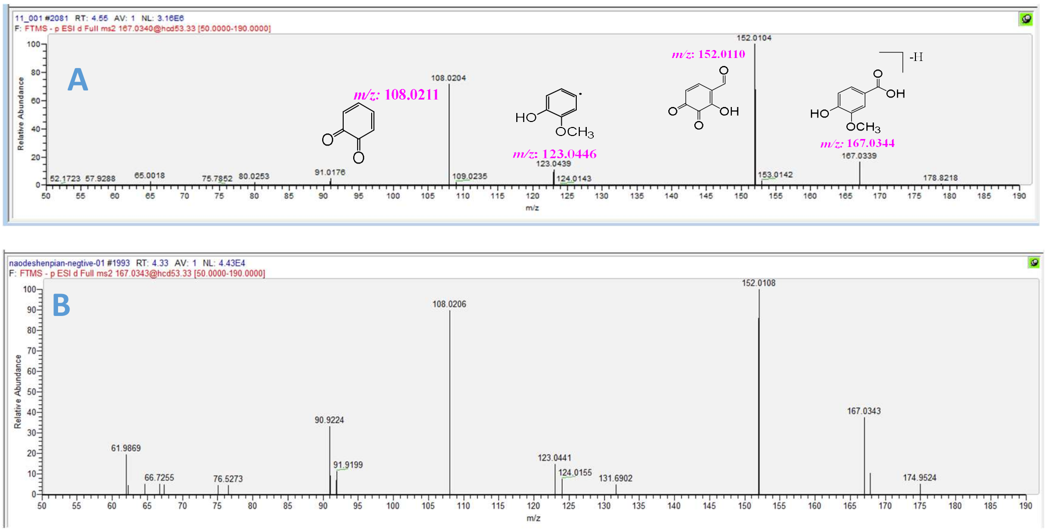

Fig. S8.1 The main results of standard vanillic acid(CAS 121-34-6, $C_8H_8O_4$)and its corresponding peak in the TIC diagram using UPLC-Q-Orbitrap-MS analysis. (**A**) The MS/MS fragments of standard vanillic acid. (**B**) The MS/MS spectra from chromatographic peak in the Naodeshengpian extract.

Note: The *m/z* values in purple are the calculated ones. The *m/z* calculation was based on the relative atomic masses of C (12.0000), H (1.007825), O (15.994915), and N (14.003074)[1].

Identification: As seen in Fig. S8.1, the R.T. value, molecular ion peak, MS/MS spectra, and characteristic pears were highly similar. Thus, the chromatographic peak in the Naodeshengpian extract was identified as vanillic acid(CAS 121-34-6, $C_8H_8O_4$).

Suppl. 9 Identification of caffeic acid CAS 331-39-5

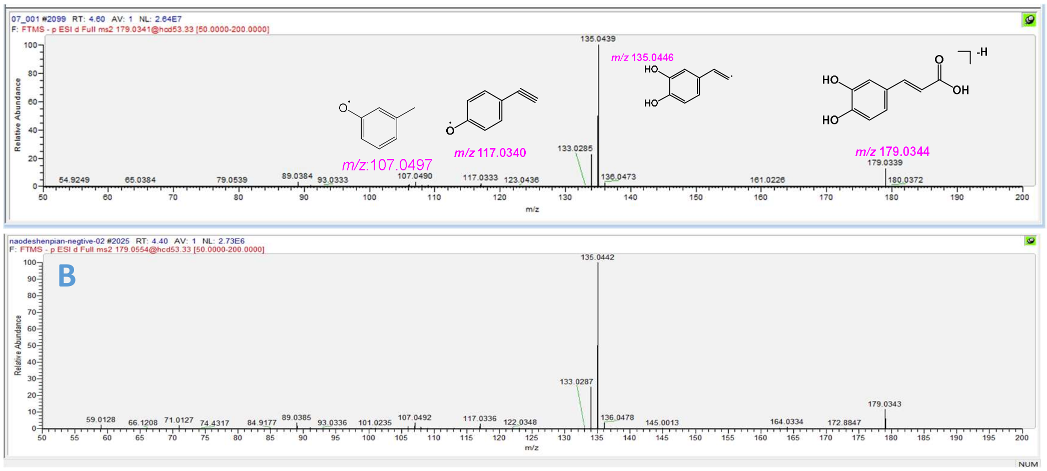

Fig. S9.1 The main results of standard caffeic acid(CAS 331-39-5, $C_9H_8O_4$)and its corresponding peak in the TIC diagram using UPLC-Q-Orbitrap-MS analysis. (**A**) The MS/MS fragments of standard caffeic acid. (**B**) The MS/MS spectra from chromatographic peak in the Naodeshengpian extract.

Note：The *m/z* values in purple are the calculated ones. The *m/z* calculation was based on the relative atomic masses of C (12.0000), H (1.007825), O (15.994915), and N (14.003074)[1].

Identification: As seen in Fig. S9.1, the R.T. value, molecular ion peak, MS/MS spectra, and characteristic pears were highly similar. Thus, the chromatographic peak in the Naodeshengpian extract was identified as caffeic acid(CAS 331-39-5, $C_9H_8O_4$).

Suppl. 10 Identification of cryptochlorogenic acid CAS 905-99-7

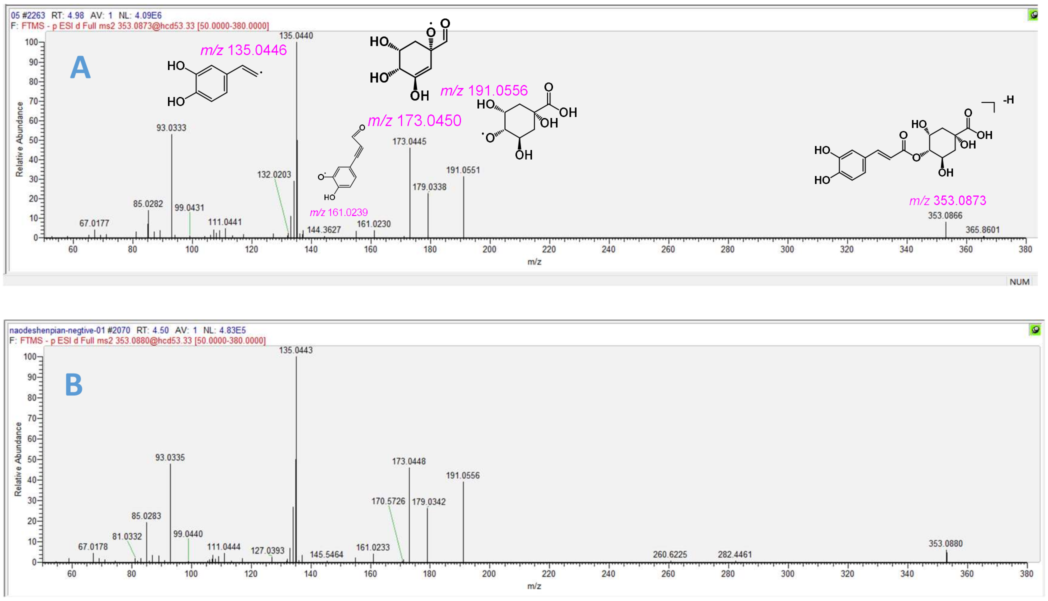

Fig. S10.1 The main results of standard cryptochlorogenic acid (CAS 905-99-7, $C_{16}H_{18}O_9$)and its corresponding peak in the TIC diagram using UPLC-Q-Orbitrap-MS analysis. (**A**) The MS/MS fragments of standard cryptochlorogenic acid. (**B**) The MS/MS spectra from chromatographic peak in the Naodeshengpian extract.

Note: The *m/z* values in purple are the calculated ones. The *m/z* calculation was based on the relative atomic masses of C (12.0000), H (1.007825), O (15.994915), and N (14.003074)[1].

Identification: As seen in Fig. S10.1, the R.T. value, molecular ion peak, MS/MS spectra, and characteristic pears were highly similar. Thus, the chromatographic peak in the Naodeshengpian extract was identified as cryptochlorogenic acid (CAS 905-99-7, $C_{16}H_{18}O_9$).

Suppl. 11 Identification of 3′-hydroxy puerarin CAS 117060-54-5

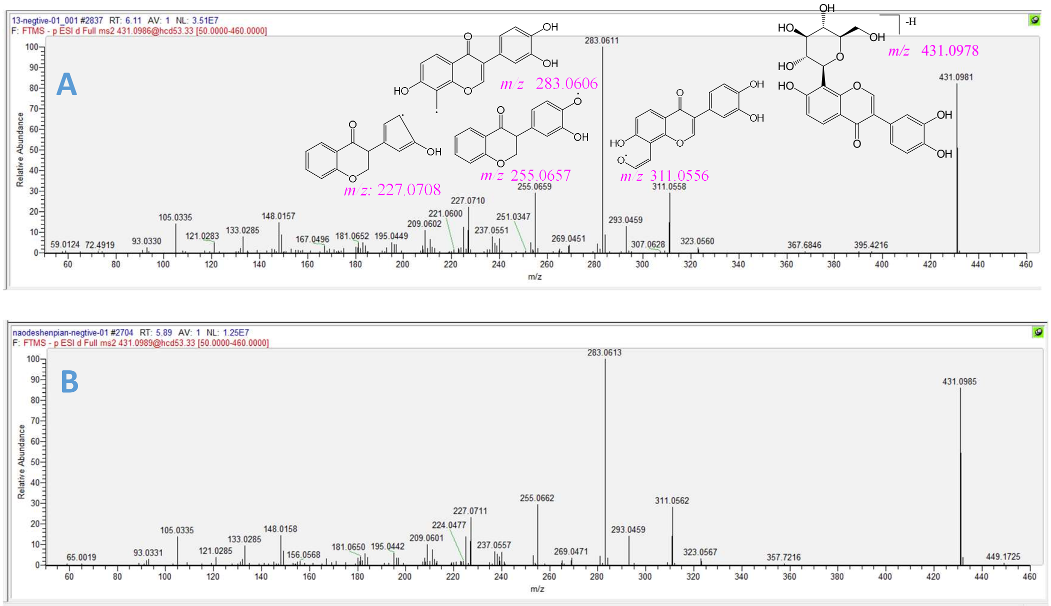

Fig. S11.1 The main results of standard 3'-hydroxy puerarin (CAS 117060-54-5, $C_{21}H_{20}O_{10}$)and its corresponding peak in the TIC diagram using UPLC-Q-Orbitrap-MS analysis. (**A**) The MS/MS fragments of standard 3'-hydroxy puerarin . (**B**) The MS/MS spectra from chromatographic peak in the Naodeshengpian extract.

Note: The *m/z* values in purple are the calculated ones. The *m/z* calculation was based on the relative atomic masses of C (12.0000), H (1.007825), O (15.994915), and N (14.003074)[1].

Identification: As seen in Fig. S11.1, the R.T. value, molecular ion peak, MS/MS spectra, and characteristic pears were highly similar. Thus, the chromatographic peak in the Naodeshengpian extract was identified as 3'-hydroxy puerarin (CAS 117060-54-5, $C_{21}H_{20}O_{10}$).

Suppl. 12 Identification of puerarin CAS 3681-99-0

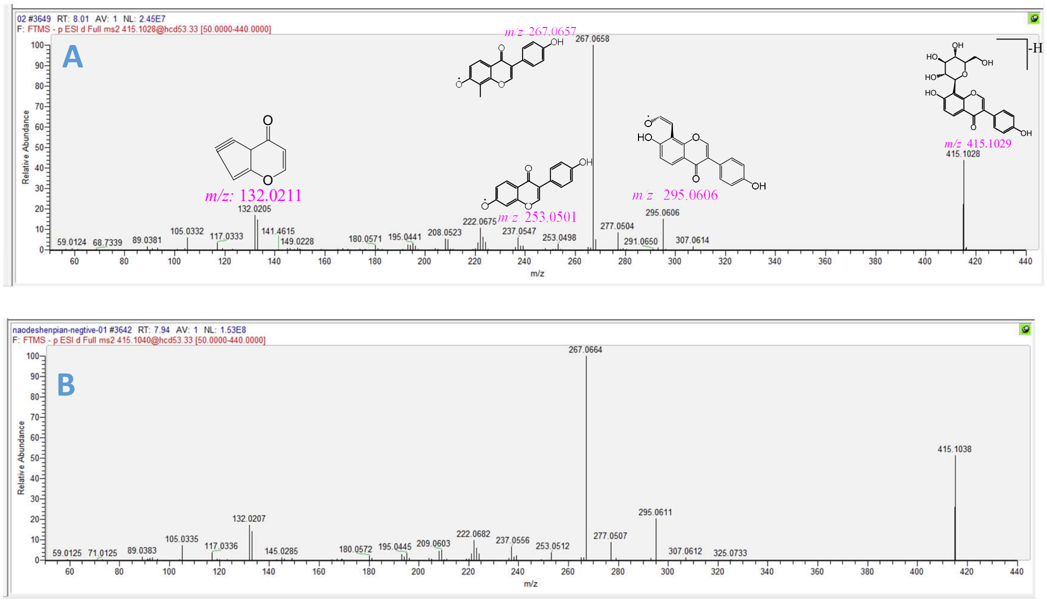

Fig. S12.1The main results of standard puerarin(CAS 3681-99-0, $C_{21}H_{20}O_9$)and its corresponding peak in the TIC diagram using UPLC-Q-Orbitrap-MS analysis. (**A**) The MS/MS fragments of standard puerarin. (**B**) The MS/MS spectra from chromatographic peak in the Naodeshengpian extract.

Note: The *m/z* values in purple are the calculated ones. The *m/z* calculation was based on the relative atomic masses of C (12.0000), H (1.007825), O (15.994915), and N (14.003074)[1].

Identification: As seen in Fig. S12.1, the R.T. value, molecular ion peak, MS/MS spectra, and characteristic pears were highly similar. Thus, the chromatographic peak in the Naodeshengpian extract was identified as puerarin(CAS 3681-99-0, $C_{21}H_{20}O_9$).

Suppl. 13 Identification of 3′-methoxy puerarin CAS 117047-07-1

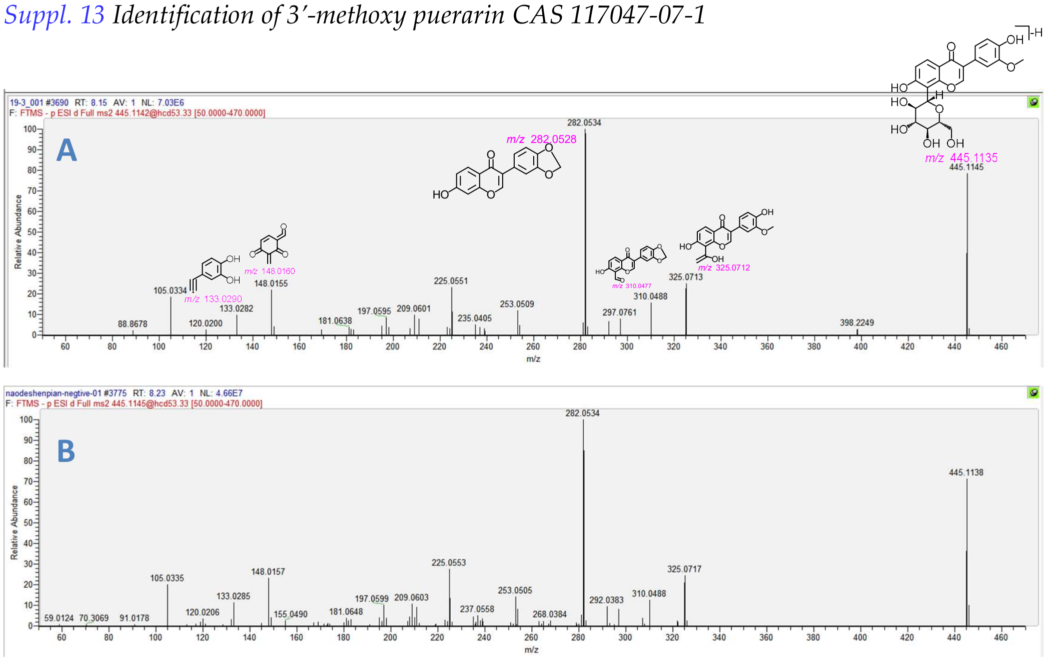

Fig. S13.1 The main results of standard 3’-methoxy puerarin(CAS 117047-07-1, $C_{22}H_{22}O_{10}$)and its corresponding peak in the TIC diagram using UPLC-Q-Orbitrap-MS analysis. (**A**) The MS/MS fragments of standard 3’-methoxy puerarin. (**B**) The MS/MS spectra from chromatographic peak in the Naodeshengpian extract.

Note: The *m/z* values in purple are the calculated ones. The *m/z* calculation was based on the relative atomic masses of C (12.0000), H (1.007825), O (15.994915), and N (14.003074)[1].

Identification: As seen in Fig. S13.1, the R.T. value, molecular ion peak, MS/MS spectra, and characteristic pears were highly similar. Thus, the chromatographic peak in the Naodeshengpian extract was identified as 3’-methoxy puerarin(CAS 117047-07-1, $C_{22}H_{22}O_{10}$).

Suppl. 14 Identification of mirificin CAS 103654-50-8

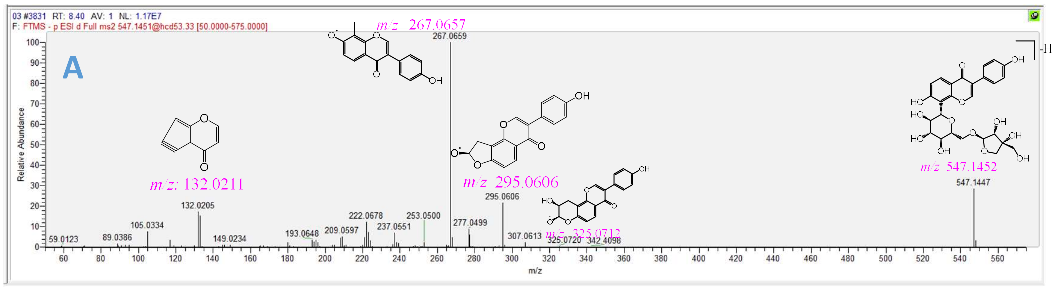

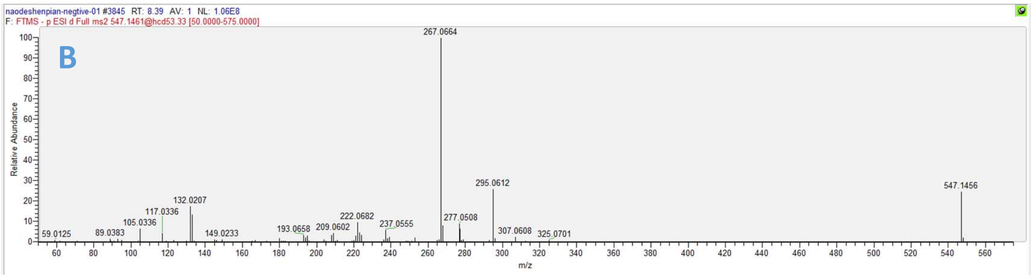

Fig. S14.1 The main results of standard mirificin (CAS 103654-50-8, $C_{26}H_{28}O_{13}$)and its corresponding peak in the TIC diagram using UPLC-Q-Orbitrap-MS analysis. (**A**) The MS/MS fragments of standard mirificin. (**B**) The MS/MS spectra from chromatographic peak in the Naodeshengpian extract.

Note: The *m/z* values in purple are the calculated ones. The *m/z* calculation was based on the relative atomic masses of C (12.0000), H (1.007825), O (15.994915), and N (14.003074)[1].

Identification: As seen in Fig. S14.1, the R.T. value, molecular ion peak, MS/MS spectra, and characteristic pears were highly similar. Thus, the chromatographic peak in the Naodeshengpian extract was identified as mirificin (CAS 103654-50-8, $C_{26}H_{28}O_{13}$).

Suppl. 15 Identification of daidzin CAS 552-66-9

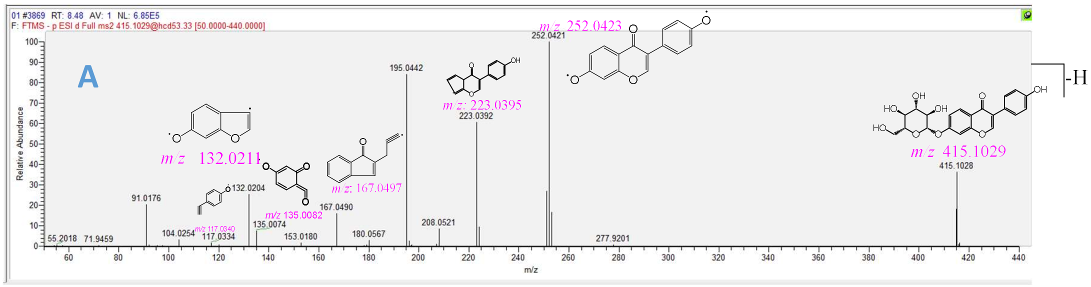

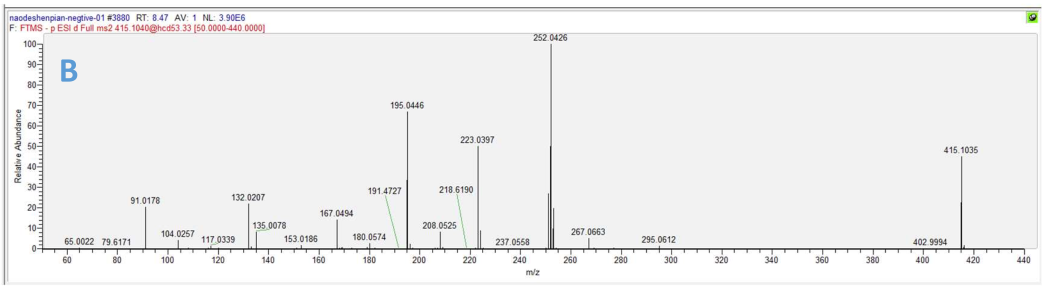

Fig. S15.1 The main results of standard daidzin (CAS 552-66-9, $C_{21}H_{20}O_9$)and its corresponding peak in the TIC diagram using UPLC-Q-Orbitrap-MS analysis. (**A**) The MS/MS fragments of standard daidzin. (**B**) The MS/MS spectra from chromatographic peak in the Naodeshengpian extract.

Note: The *m/z* values in purple are the calculated ones. The *m/z* calculation was based on the relative atomic masses of C (12.0000), H (1.007825), O (15.994915), and N (14.003074)[1].

Identification: As seen in Fig. S15.1, the R.T. value, molecular ion peak, MS/MS spectra, and characteristic pears were highly similar. Thus, the chromatographic peak in the Naodeshengpian extract was identified as daidzin (CAS 552-66-9, $C_{21}H_{20}O_9$).

Suppl. 16 Identification of ferulic acid CAS 1135-24-6

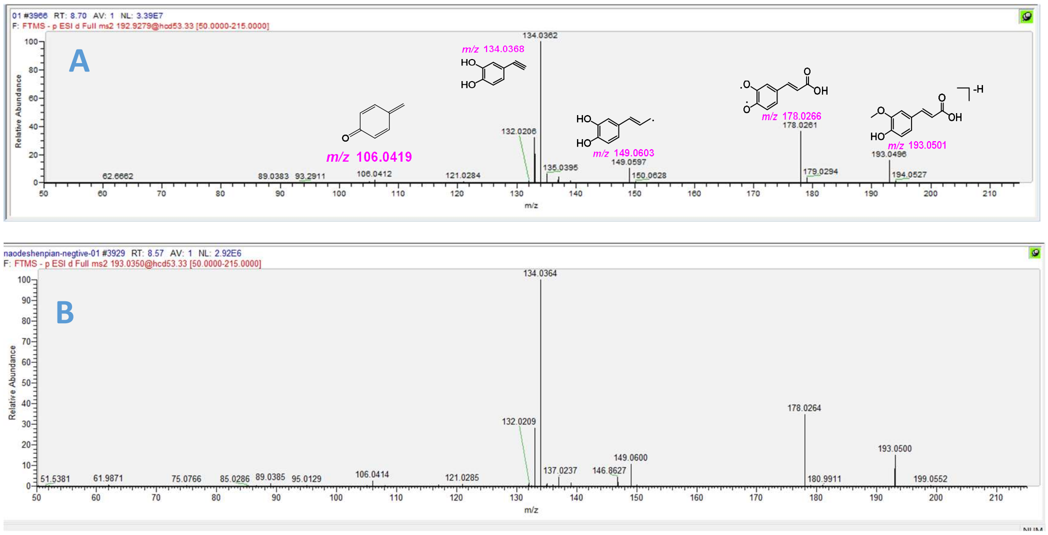

Fig. S16.1 The main results of standard ferulic acid (CAS 1135-24-6, $C_{10}H_{10}O_4$)and its corresponding peak in the TIC diagram using UPLC-Q-Orbitrap-MS analysis. (**A**) The MS/MS fragments of standard ferulic acid. (**B**) The MS/MS spectra from chromatographic peak in the Naodeshengpian extract.

Note: The *m/z* values in purple are the calculated ones. The *m/z* calculation was based on the relative atomic masses of C (12.0000), H (1.007825), O (15.994915), and N (14.003074)[1].

Identification: As seen in Fig. S16.1, the R.T. value, molecular ion peak, MS/MS spectra, and characteristic pears were highly similar. Thus, the chromatographic peak in the Naodeshengpian extract was identified as ferulic acid (CAS 1135-24-6, $C_{10}H_{10}O_4$).

Suppl. 17 Identification of isoferulic acid CAS 537-73-5

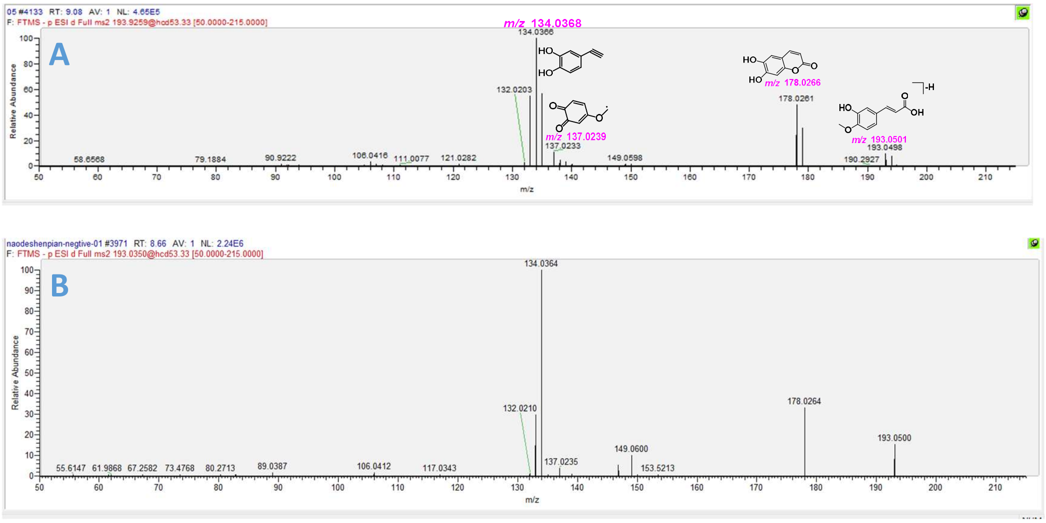

Fig. S17.1 The main results of standard isoferulic acid(CAS 537-73-5, $C_{10}H_{10}O_4$)and its corresponding peak in the TIC diagram using UPLC-Q-Orbitrap-MS analysis. (**A**) The MS/MS fragments of standard isoferulic acid. (**B**) The MS/MS spectra from chromatographic peak in the Naodeshengpian extract.

Note: The *m/z* values in purple are the calculated ones. The *m/z* calculation was based on the relative atomic masses of C (12.0000), H (1.007825), O (15.994915), and N (14.003074)[1].

Identification: As seen in Fig. S17.1, the R.T. value, molecular ion peak, MS/MS spectra, and characteristic pears were highly similar. Thus, the chromatographic peak in the Naodeshengpian extract was identified as isoferulic acid(CAS 537-73-5, $C_{10}H_{10}O_4$).

Suppl. 18 Identification of glycitin CAS 40246-10-4

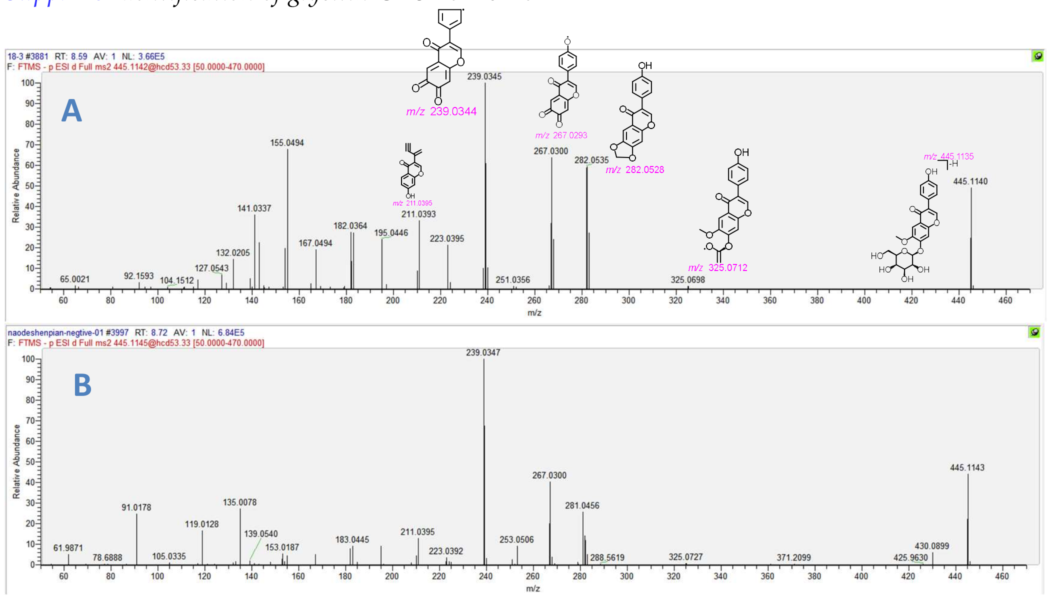

Fig. S18.1 The main results of standard glycitin(CAS 40246-10-4, $C_{22}H_{22}O_{10}$)and its corresponding peak in the TIC diagram using UPLC-Q-Orbitrap-MS analysis. (**A**) The MS/MS fragments of standard glycitin. (**B**) The MS/MS spectra from chromatographic peak in the Naodeshengpian extract.

Note: The *m/z* values in purple are the calculated ones. The *m/z* calculation was based on the relative atomic masses of C (12.0000), H (1.007825), O (15.994915), and N (14.003074)[1].

Identification: As seen in Fig. S18.1, the R.T. value, molecular ion peak, MS/MS spectra, and characteristic pears were highly similar. Thus, the chromatographic peak in the Naodeshengpian extract was identified as glycitin(CAS 40246-10-4, $C_{22}H_{22}O_{10}$).

Suppl. 19 Identification of genistin CAS 529-59-9

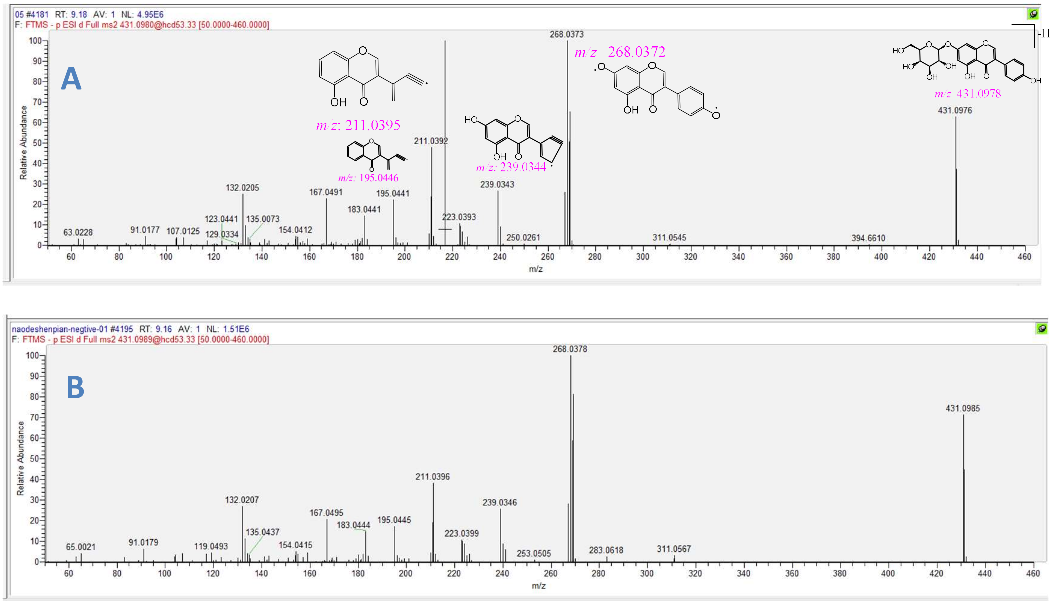

Fig. S19.1 The main results of standard genistin(CAS 529-59-9, $C_{21}H_{20}O_{10}$)and its corresponding peak in the TIC diagram using UPLC-Q-Orbitrap-MS analysis. (**A**) The MS/MS fragments of standard genistin. (**B**) The MS/MS spectra from chromatographic peak in the Naodeshengpian extract.

Note: The *m/z* values in purple are the calculated ones. The *m/z* calculation was based on the relative atomic masses of C (12.0000), H (1.007825), O (15.994915), and N (14.003074)[1].

Identification: As seen in Fig. S19.1, the R.T. value, molecular ion peak, MS/MS spectra, and characteristic pears were highly similar. Thus, the chromatographic peak in the Naodeshengpian extract was identified as genistin(CAS 529-59-9, $C_{21}H_{20}O_{10}$).

Suppl. 20 Identification of 4-methyl-2,6-dimethoxyphenol CAS 6638-05-7

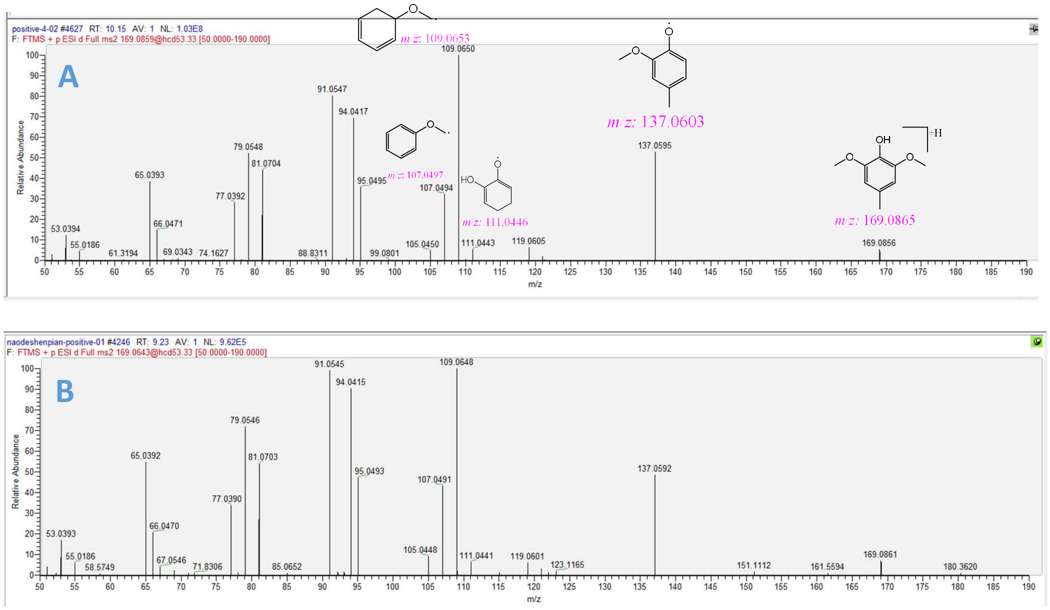

Fig. S20.1 The main results of standard 4-methyl-2,6-dimethoxyphenol(CAS 6638-05-7, $C_9H_{12}O_3$)and its corresponding peak in the TIC diagram using UPLC-Q-Orbitrap-MS analysis. (**A**) The MS/MS fragments of standard 4-methyl-2,6-dimethoxyphenol. (**B**) The MS/MS spectra from chromatographic peak in the Naodeshengpian extract.

Note: The *m/z* values in purple are the calculated ones. The *m/z* calculation was based on the relative atomic masses of C (12.0000), H (1.007825), O (15.994915), and N (14.003074)[1].

Identification: As seen in Fig. S20.1, the R.T. value, molecular ion peak, MS/MS spectra, and characteristic pears were highly similar. Thus, the chromatographic peak in the Naodeshengpian extract was identified as 4-methyl-2,6-dimethoxyphenol(CAS 6638-05-7, $C_9H_{12}O_3$).

Suppl. 21 Identification of hyperoside CAS 482-36-0

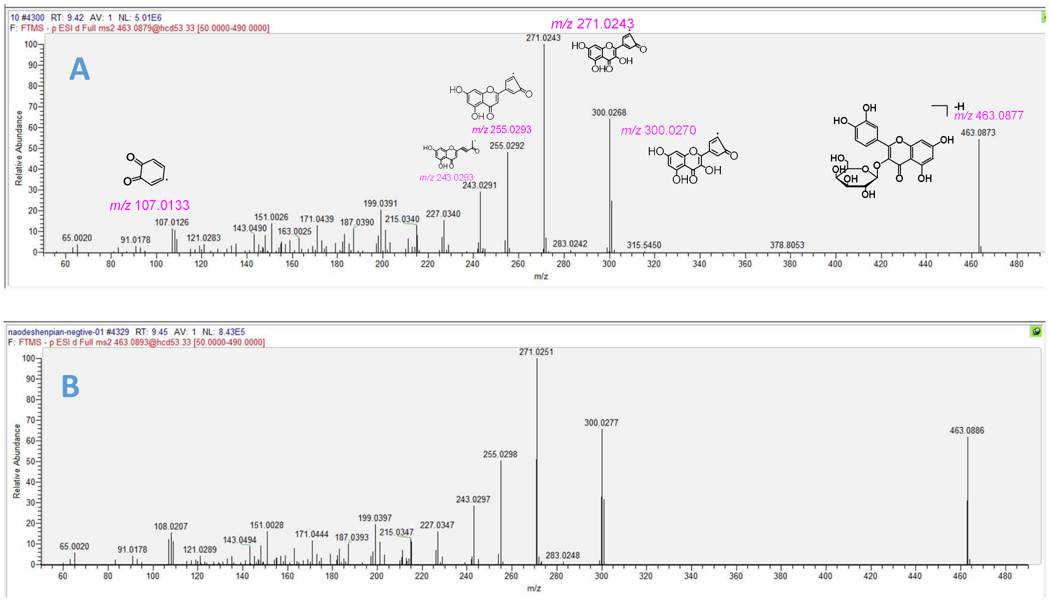

Fig. S21.1 The main results of standard hyperoside(CAS 482-36-0, $C_{21}H_{20}O_{12}$)and its corresponding peak in the TIC diagram using UPLC-Q-Orbitrap-MS analysis. (**A**) The MS/MS fragments of standard hyperoside. (**B**) The MS/MS spectra from chromatographic peak in the Naodeshengpian extract.

Note: The *m/z* values in purple are the calculated ones. The *m/z* calculation was based on the relative atomic masses of C (12.0000), H (1.007825), O (15.994915), and N (14.003074)[1].

Identification: As seen in Fig. S21.1, the R.T. value, molecular ion peak, MS/MS spectra, and characteristic pears were highly similar. Thus, the chromatographic peak in the Naodeshengpian extract was identified as hyperoside(CAS 482-36-0, $C_{21}H_{20}O_{12}$).

Suppl. 22 Identification of rutin CAS 153-18-4

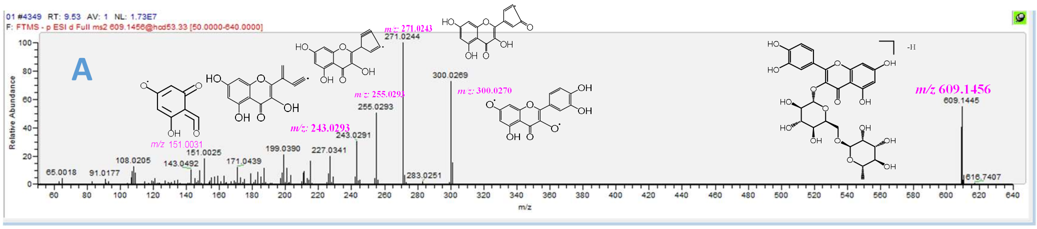

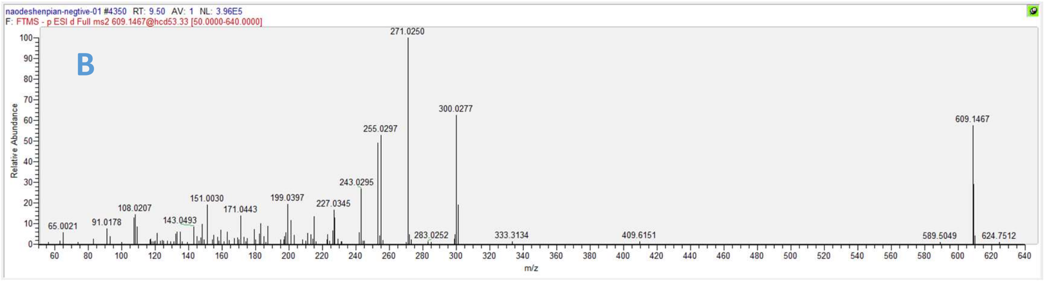

Fig. S22.1 The main results of standard rutin(CAS 153-18-4, $C_{27}H_{30}O_{16}$)and its corresponding peak in the TIC diagram using UPLC-Q-Orbitrap-MS analysis. (**A**) The MS/MS fragments of standard rutin. (**B**) The MS/MS spectra from chromatographic peak in the Naodeshengpian extract.

Note: The *m/z* values in purple are the calculated ones. The *m/z* calculation was based on the relative atomic masses of C (12.0000), H (1.007825), O (15.994915), and N (14.003074)[1].

Identification: As seen in Fig. S22.1, the R.T. value, molecular ion peak, MS/MS spectra, and characteristic pears were highly similar. Thus, the chromatographic peak in the Naodeshengpian extract was identified as rutin(CAS 153-18-4, $C_{27}H_{30}O_{16}$).

Suppl. 23 Identification of isoquercitrin CAS 482-35-9

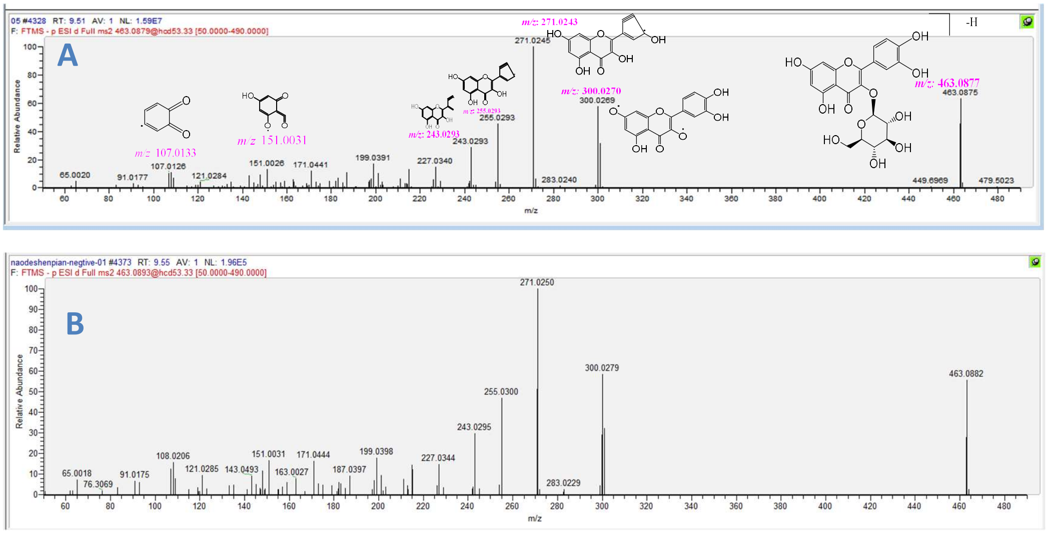

Fig. S23.1 The main results of standard isoquercitrin(CAS 482-35-9, $C_{21}H_{20}O_{12}$)and its corresponding peak in the TIC diagram using UPLC-Q-Orbitrap-MS analysis. (**A**) The MS/MS fragments of standard isoquercitrin. (**B**) The MS/MS spectra from chromatographic peak in the Naodeshengpian extract.

Note：The *m/z* values in purple are the calculated ones. The *m/z* calculation was based on the relative atomic masses of C (12.0000), H (1.007825), O (15.994915), and N (14.003074)[1].

Identification: As seen in Fig. S23.1, the R.T. value, molecular ion peak, MS/MS spectra, and characteristic pears were highly similar. Thus, the chromatographic peak in the Naodeshengpian extract was identified as Isoquercitrin(CAS 482-35-9, $C_{21}H_{20}O_{12}$).

Suppl. 24 Identification of naringin CAS 10236-47-2

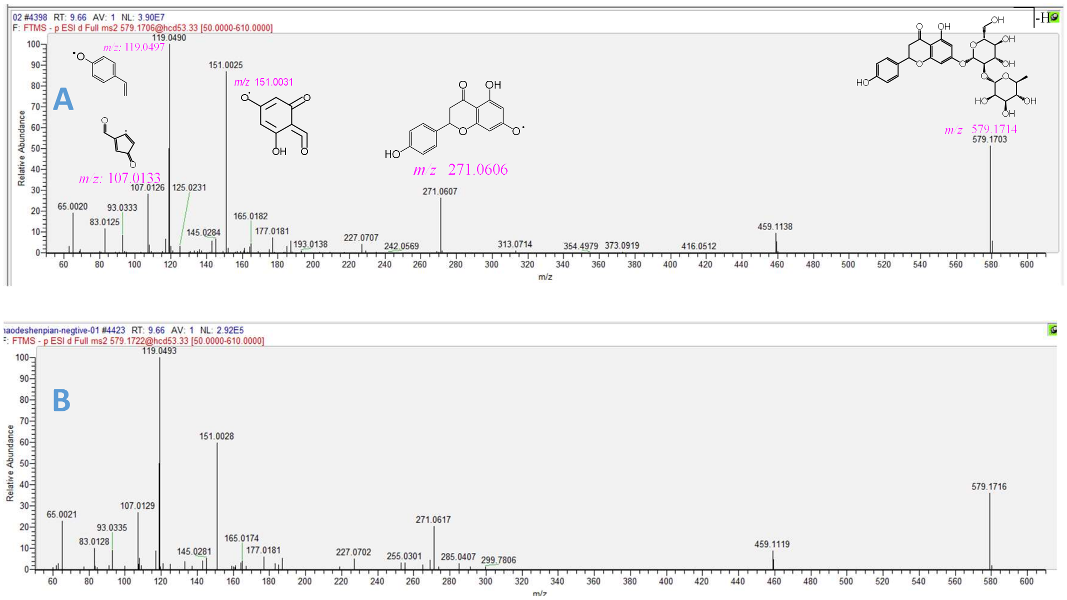

Fig. S24.1 The main results of standard naringin(CAS 10236-47-2, $C_{27}H_{32}O_{14}$)and its corresponding peak in the TIC diagram using UPLC-Q-Orbitrap-MS analysis. (**A**) The MS/MS fragments of standard naringin. (**B**) The MS/MS spectra from chromatographic peak in the Naodeshengpian extract.

Note: The *m/z* values in purple are the calculated ones. The *m/z* calculation was based on the relative atomic masses of C (12.0000), H (1.007825), O (15.994915), and N (14.003074)[1].

Identification: As seen in Fig. S24.1, the R.T. value, molecular ion peak, MS/MS spectra, and characteristic pears were highly similar. Thus, the chromatographic peak in the Naodeshengpian extract was identified as naringin(CAS 10236-47-2, $C_{27}H_{32}O_{14}$).

Suppl. 25 Identification of cosmosiin CAS 578-74-5

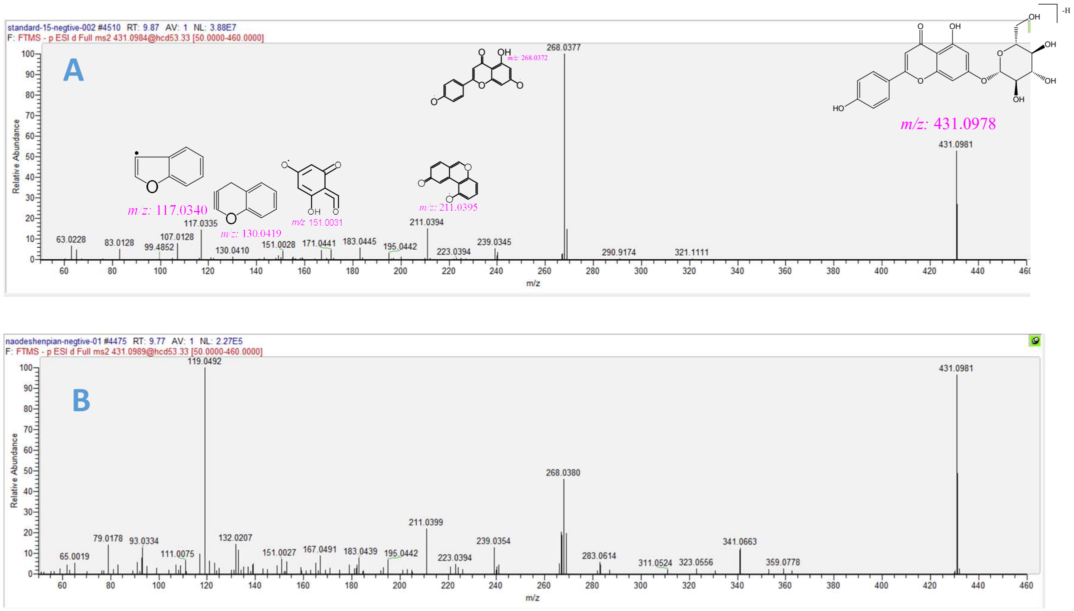

Fig. S25.1 The main results of standard cosmosiin(CAS 578-74-5, $C_{21}H_{20}O_{10}$)and its corresponding peak in the TIC diagram using UPLC-Q-Orbitrap-MS analysis. (**A**) The MS/MS fragments of standard cosmosiin. (**B**) The MS/MS spectra from chromatographic peak in the Naodeshengpian extract.

Note: The *m/z* values in purple are the calculated ones. The *m/z* calculation was based on the relative atomic masses of C (12.0000), H (1.007825), O (15.994915), and N (14.003074)[1].

Identification: As seen in Fig. S25.1, the R.T. value, molecular ion peak, MS/MS spectra, and characteristic pears were highly similar. Thus, the chromatographic peak in the Naodeshengpian extract was identified as cosmosiin(CAS 578-74-5, $C_{21}H_{20}O_{10}$).

Suppl. 26 Identification of astragalin CAS 480-10-4

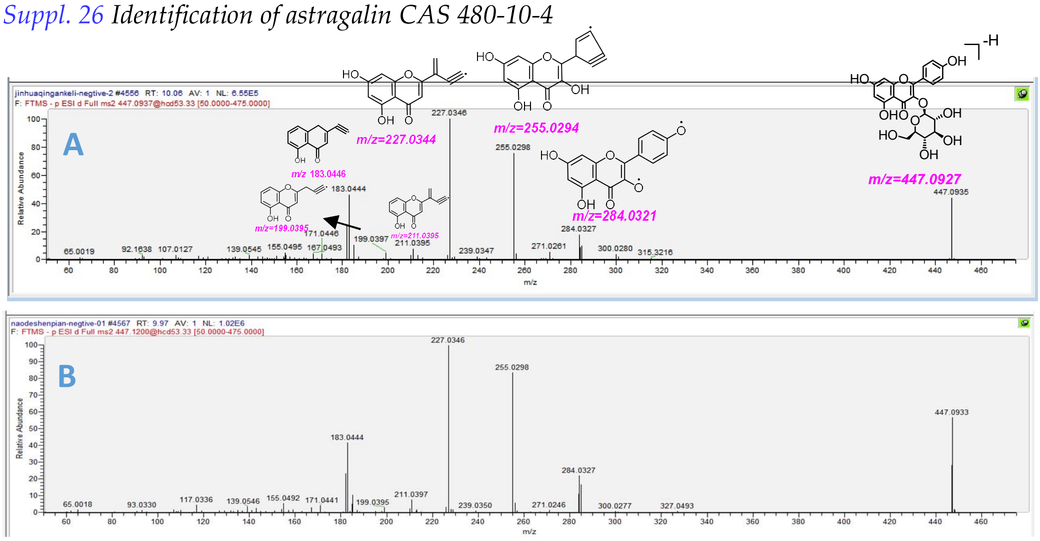

Fig. S26.1 The main results of standard astragalin(CAS 480-10-4, $C_{21}H_{20}O_{11}$)and its corresponding peak in the TIC diagram using UPLC-Q-Orbitrap-MS analysis. (**A**) The MS/MS fragments of standard astragalin. (**B**) The MS/MS spectra from chromatographic peak in the Naodeshengpian extract.

Note：The *m/z* values in purple are the calculated ones. The *m/z* calculation was based on the relative atomic masses of C (12.0000), H (1.007825), O (15.994915), and N (14.003074)[1].

Identification: As seen in Fig. S26.1, the R.T. value, molecular ion peak, MS/MS spectra, and characteristic pears were highly similar. Thus, the chromatographic peak in the Naodeshengpian extract was identified as astragalin(CAS 480-10-4, $C_{21}H_{20}O_{11}$).

Suppl. 27 Identification of 2′-hydroxygenistein CAS 1156-78-1

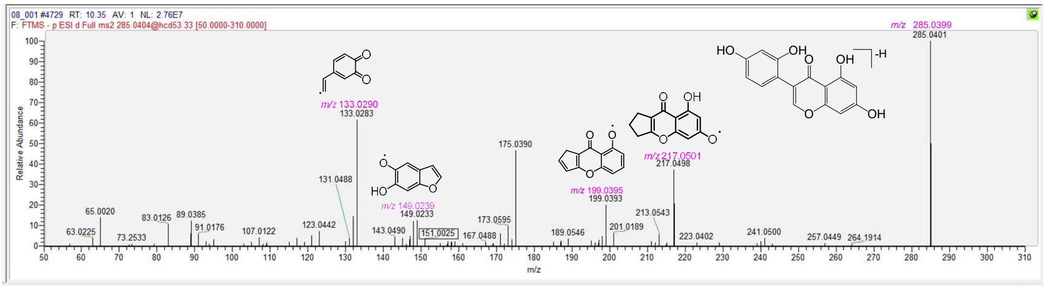

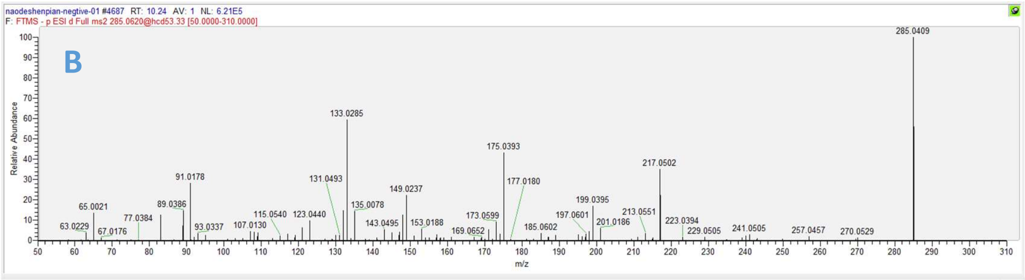

Fig. S27.1 The main results of standard 2'-hydroxygenistein(CAS 1156-78-1, $C_{15}H_{10}O_6$)and its corresponding peak in the TIC diagram using UPLC-Q-Orbitrap-MS analysis. (**A**) The MS/MS fragments of standard 2'-hydroxygenistein. (**B**) The MS/MS spectra from chromatographic peak in the Naodeshengpian extract.

Note: The *m/z* values in purple are the calculated ones. The *m/z* calculation was based on the relative atomic masses of C (12.0000), H (1.007825), O (15.994915), and N (14.003074)[1].

Identification: As seen in Fig. S27.1, the R.T. value, molecular ion peak, MS/MS spectra, and characteristic pears were highly similar. Thus, the chromatographic peak in the Naodeshengpian extract was identified as 2'-hydroxygenistein(CAS 1156-78-1, $C_{15}H_{10}O_6$).

Suppl. 28 Identification of daidzein CAS 486-66-8

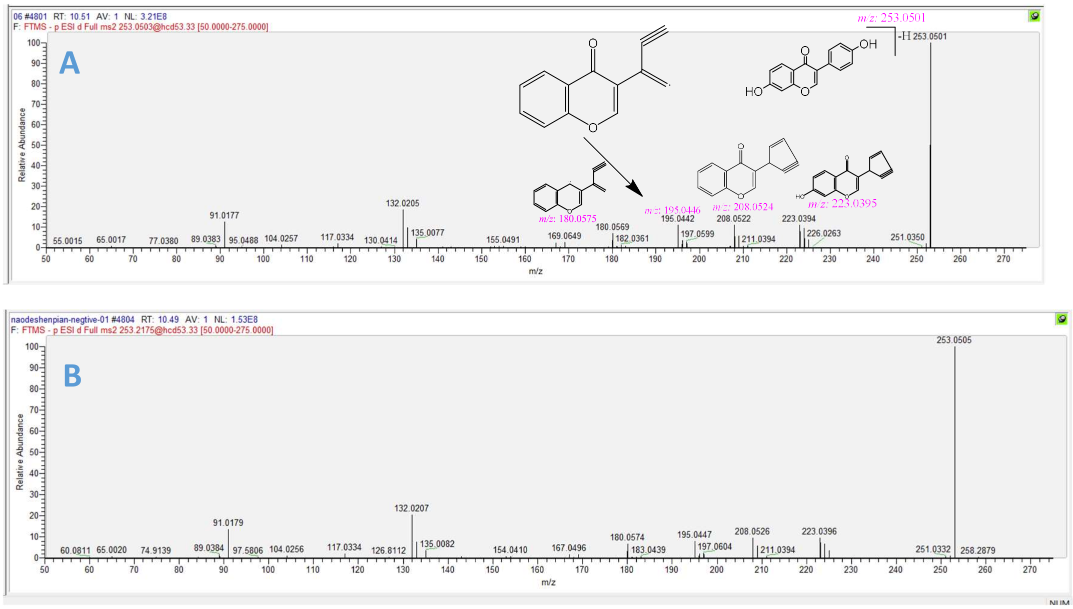

Fig. S28.1 The main results of standard daidzein(CAS 486-66-8, $C_{15}H_{10}O_4$)and its corresponding peak in the TIC diagram using UPLC-Q-Orbitrap-MS analysis. (**A**) The MS/MS fragments of standard daidzein. (**B**) The MS/MS spectra from chromatographic peak in the Naodeshengpian extract.

Note: The *m/z* values in purple are the calculated ones. The *m/z* calculation was based on the relative atomic masses of C (12.0000), H (1.007825), O (15.994915), and N (14.003074)[1].

Identification: As seen in Fig. S28.1, the R.T. value, molecular ion peak, MS/MS spectra, and characteristic pears were highly similar. Thus, the chromatographic peak in the Naodeshengpian extract was identified as daidzein(CAS486-66-8, $C_{15}H_{10}O_4$).

Suppl. 29 Identification of calycosin CAS 20575-57-9

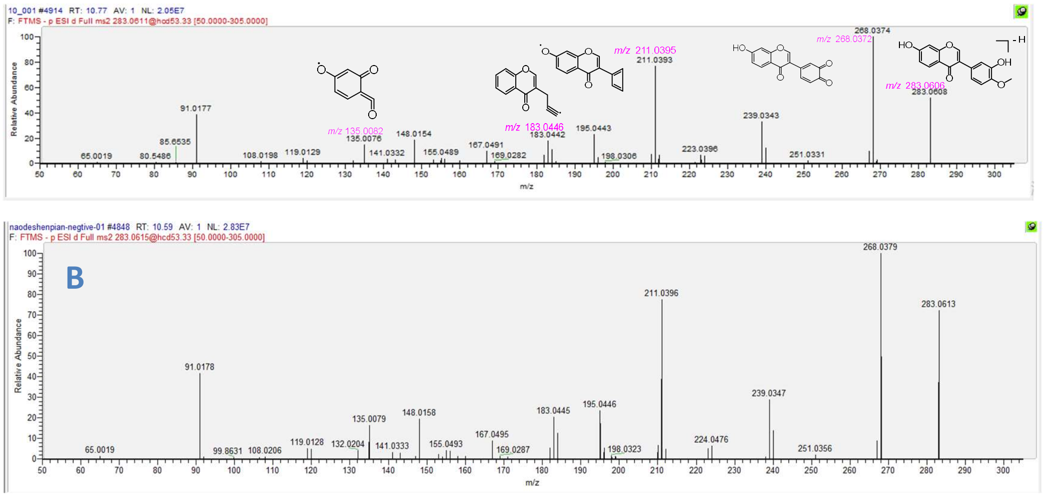

Fig. S29.1 The main results of standard calycosin(CAS 20575-57-9, $C_{16}H_{12}O_5$)and its corresponding peak in the TIC diagram using UPLC-Q-Orbitrap-MS analysis. (**A**) The MS/MS fragments of standard calycosin. (**B**) The MS/MS spectra from chromatographic peak in the Naodeshengpian extract.

Note: The *m/z* values in purple are the calculated ones. The *m/z* calculation was based on the relative atomic masses of C (12.0000), H (1.007825), O (15.994915), and N (14.003074)[1].

Identification: As seen in Fig. S29.1, the R.T. value, molecular ion peak, MS/MS spectra, and characteristic pears were highly similar. Thus, the chromatographic peak in the Naodeshengpian extract was identified as calycosin(CAS 20575-57-9, $C_{16}H_{12}O_5$).

Suppl. 30 Identification of quercetin CAS 117-39-5

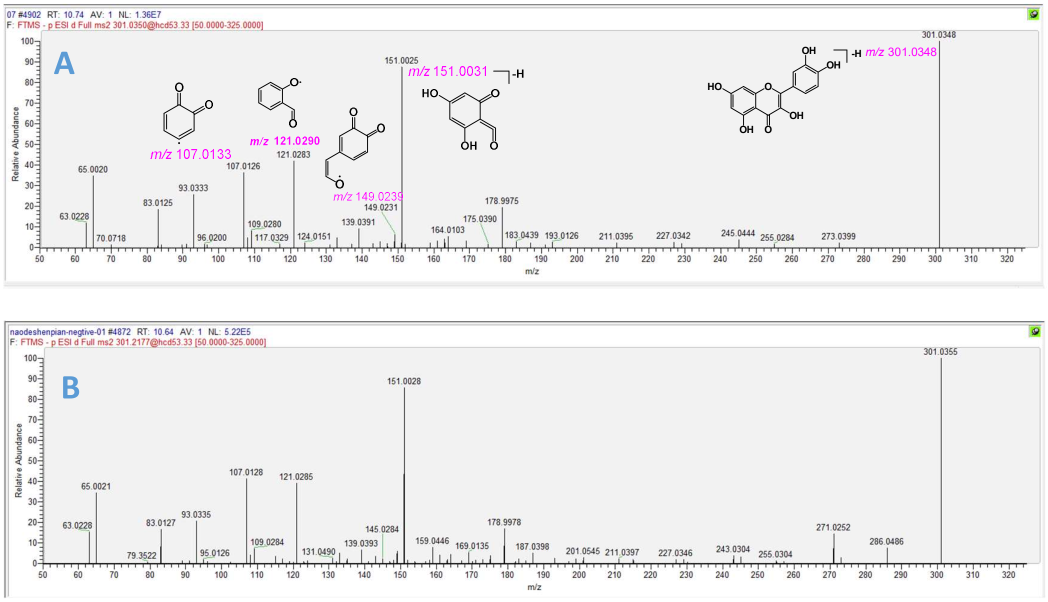

Fig. S30.1 The main results of standard quercetin(CAS 117-39-5, $C_{15}H_{10}O_7$)and its corresponding peak in the TIC diagram using UPLC-Q-Orbitrap-MS analysis. (**A**) The MS/MS fragments of standard quercetin. (**B**) The MS/MS spectra from chromatographic peak in the Naodeshengpian extract.

Note: The *m/z* values in purple are the calculated ones. The *m/z* calculation was based on the relative atomic masses of C (12.0000), H (1.007825), O (15.994915), and N (14.003074)[1].

Identification: As seen in Fig. S30.1, the R.T. value, molecular ion peak, MS/MS spectra, and characteristic pears were highly similar. Thus, the chromatographic peak in the Naodeshengpian extract was identified as quercetin(CAS 117-39-5, $C_{15}H_{10}O_7$).

Suppl. 31 Identification of 7,4′-dihydroxyflavone CAS 2196-14-7

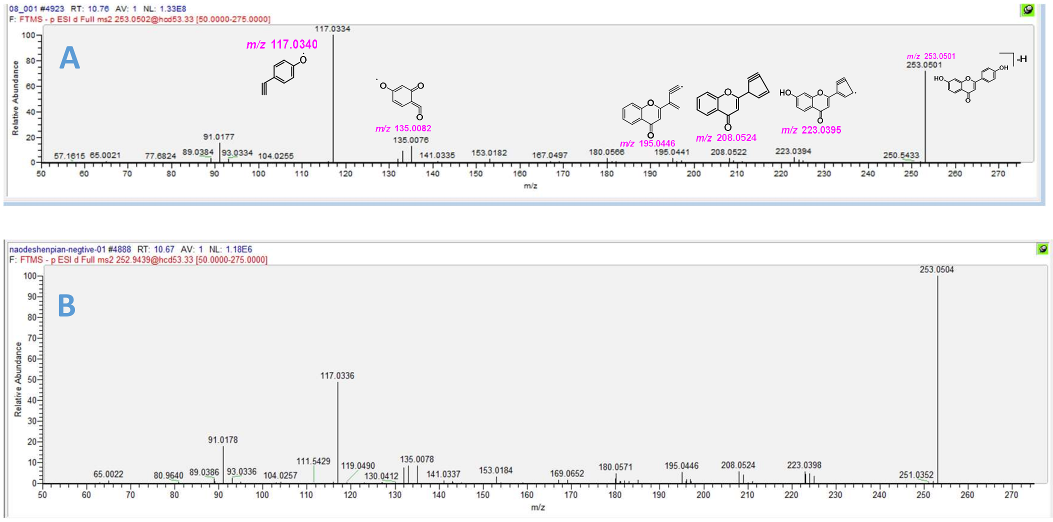

Fig. S31.1 The main results of standard 7,4'-dihydroxyflavone(CAS 2196-14-7, $C_{15}H_{10}O_4$)and its corresponding peak in the TIC diagram using UPLC-Q-Orbitrap-MS analysis. (**A**) The MS/MS fragments of standard 7,4'-dihydroxyflavone. (**B**) The MS/MS spectra from chromatographic peak in the Naodeshengpian extract.

Note: The *m/z* values in purple are the calculated ones. The *m/z* calculation was based on the relative atomic masses of C (12.0000), H (1.007825), O (15.994915), and N (14.003074)[1].

Identification: As seen in Fig. S31.1, the R.T. value, molecular ion peak, MS/MS spectra, and characteristic pears were highly similar. Thus, the chromatographic peak in the Naodeshengpian extract was identified as 7,4'-dihydroxyflavone(CAS 2196-14-7, $C_{15}H_{10}O_4$).

Suppl. 32 Identification of syringic acid CAS 530-57-4

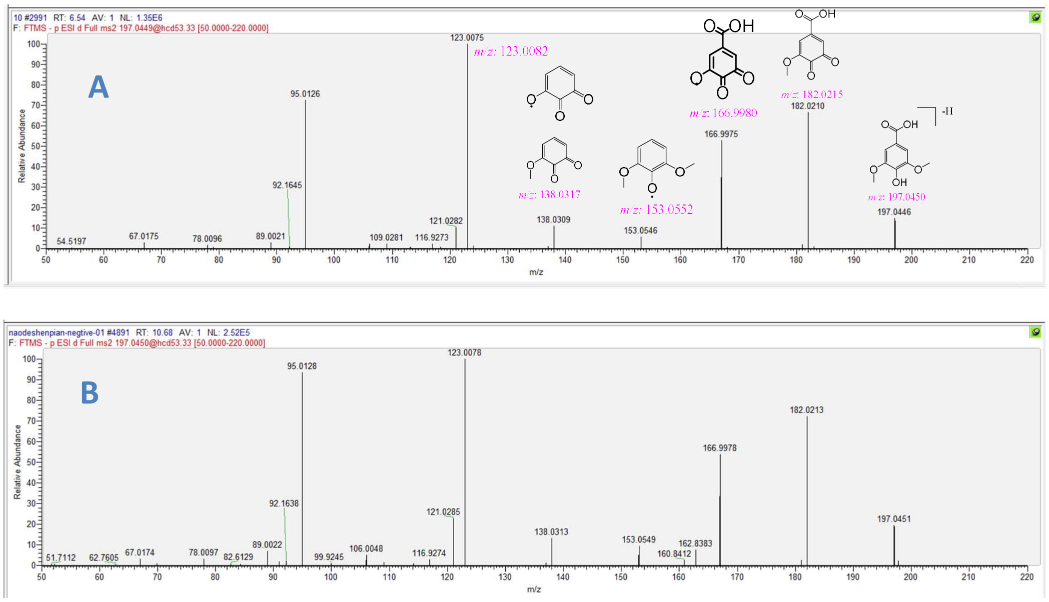

Fig. S32.1 The main results of standard syringic acid(CAS 530-57-4, $C_9H_{10}O_5$)and its corresponding peak in the TIC diagram using UPLC-Q-Orbitrap-MS analysis. (**A**) The MS/MS fragments of standard syringic acid. (**B**) The MS/MS spectra from chromatographic peak in the Naodeshengpian extract.

Note: The *m/z* values in purple are the calculated ones. The *m/z* calculation was based on the relative atomic masses of C (12.0000), H (1.007825), O (15.994915), and N (14.003074)[1].

Identification: As seen in Fig. S32.1, the R.T. value, molecular ion peak, MS/MS spectra, and characteristic pears were highly similar. Thus, the chromatographic peak in the Naodeshengpian extract was identified as syringic acid(CAS 530-57-4, $C_9H_{10}O_5$).

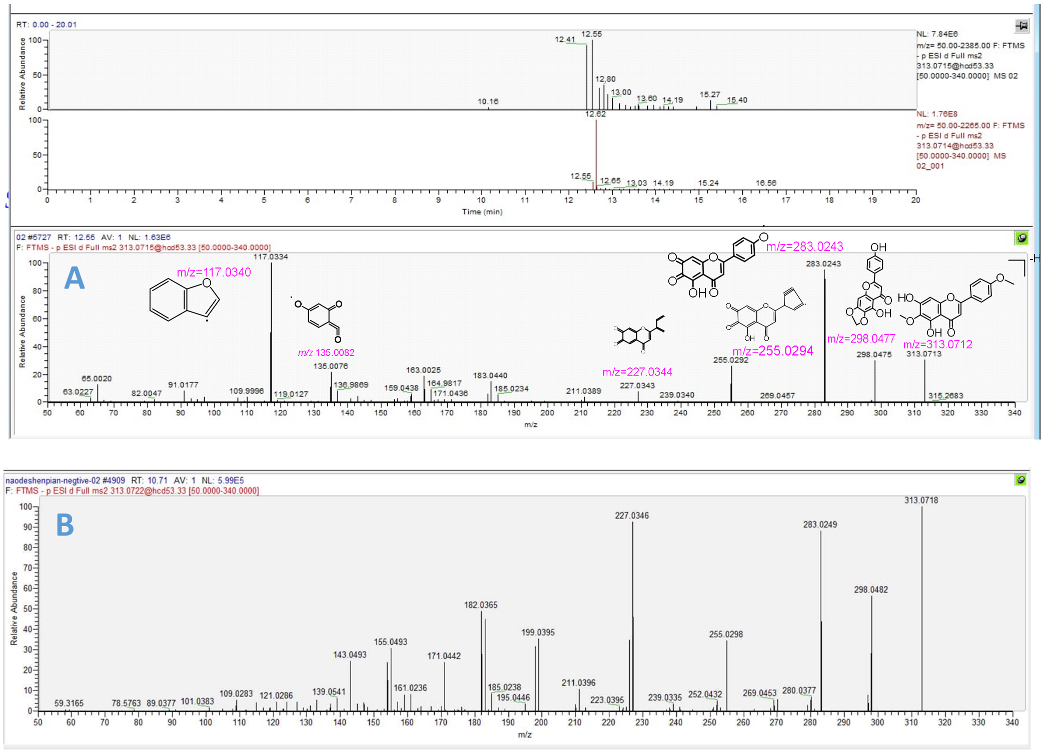

Fig. S33.1 The main results of standard pectolinarigenin(CAS 520-12-7, $C_{17}H_{14}O_6$)and its corresponding peak in the TIC diagram using UPLC-Q-Orbitrap-MS analysis. (**A**) The MS/MS fragments of standard pectolinarigenin. (**B**) The MS/MS spectra from chromatographic peak in the Naodeshengpian extract.

Note: The *m/z* values in purple are the calculated ones. The *m/z* calculation was based on the relative atomic masses of C (12.0000), H (1.007825), O (15.994915), and N (14.003074)[1].

Identification: As seen in Fig. S33.1, the R.T. value, molecular ion peak, MS/MS spectra, and characteristic pears were highly similar. Thus, the chromatographic peak in the Naodeshengpian extract was identified as pectolinarigenin(CAS 520-12-7, $C_{17}H_{14}O_6$).

Suppl. 34 Identification of luteolin CAS 491-70-3

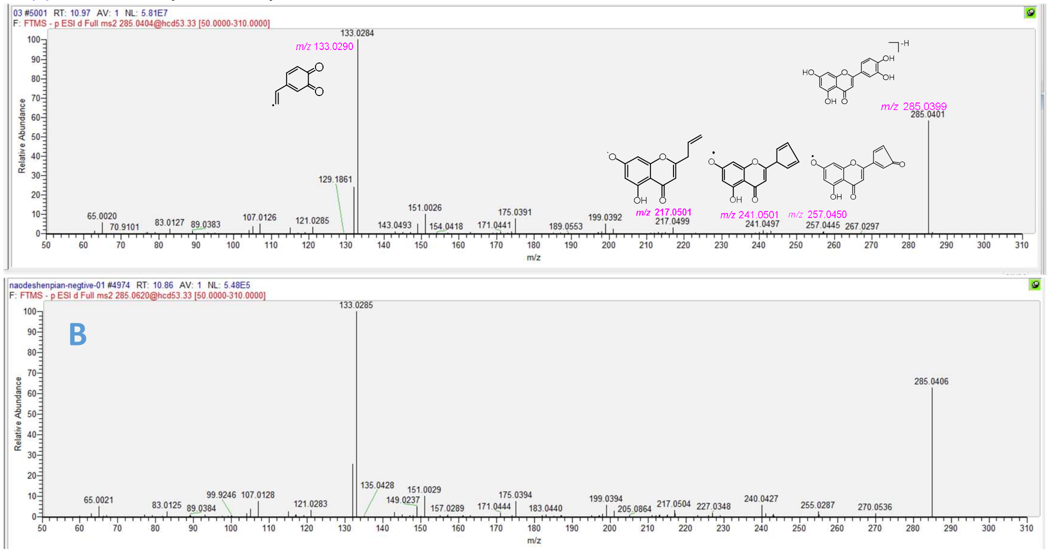

Fig. S34.1 The main results of standard luteolin(CAS 491-70-3, $C_{15}H_{10}O_6$)and its corresponding peak in the TIC diagram using UPLC-Q-Orbitrap-MS analysis. (**A**) The MS/MS fragments of standard luteolin. (**B**) The MS/MS spectra from chromatographic peak in the Naodeshengpian extract.

Note: The *m/z* values in purple are the calculated ones. The *m/z* calculation was based on the relative atomic masses of C (12.0000), H (1.007825), O (15.994915), and N (14.003074)[1].

Identification: As seen in Fig. S34.1, the R.T. value, molecular ion peak, MS/MS spectra, and characteristic pears were highly similar. Thus, the chromatographic peak in the Naodeshengpian extract was identified as luteolin(CAS 491-70-3, $C_{15}H_{10}O_6$).

Suppl. 35 Identification of genistein CAS 446-72-0

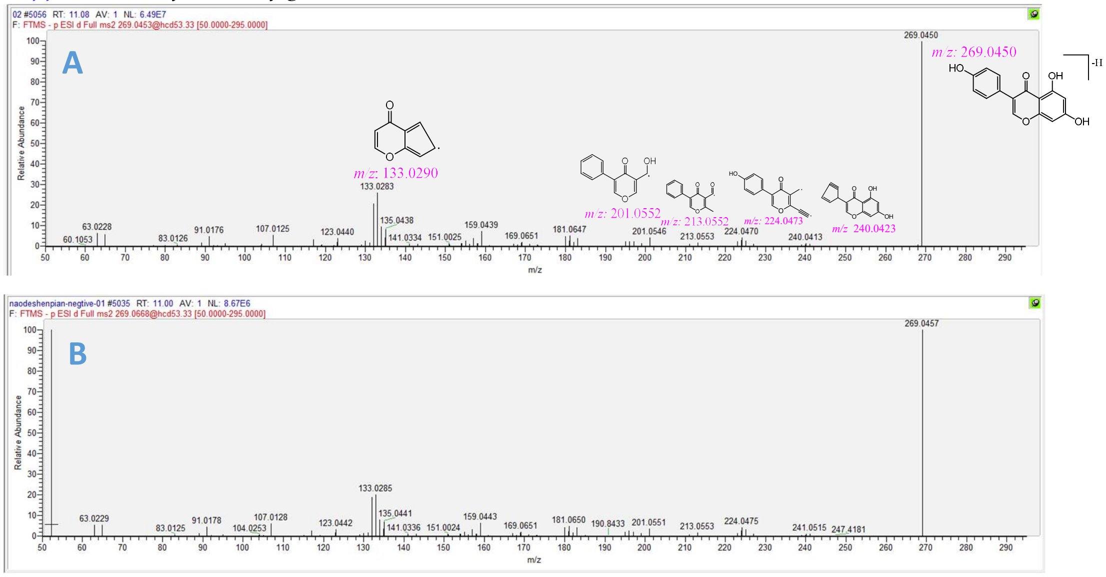

Fig. S35.1 The main results of standard genistein(CAS 446-72-0, $C_{15}H_{10}O_5$)and its corresponding peak in the TIC diagram using UPLC-Q-Orbitrap-MS analysis. (**A**) The MS/MS fragments of standard genistein. (**B**) The MS/MS spectra from chromatographic peak in the Naodeshengpian extract.

Note: The *m/z* values in purple are the calculated ones. The *m/z* calculation was based on the relative atomic masses of C (12.0000), H (1.007825), O (15.994915), and N (14.003074)[1].

Identification: As seen in Fig. S35.1, the R.T. value, molecular ion peak, MS/MS spectra, and characteristic pears were highly similar. Thus, the chromatographic peak in the Naodeshengpian extract was identified as genistein(CAS 446-72-0, $C_{15}H_{10}O_5$).

Suppl. 36 Identification of notoginsenoside R1 CAS 80418-24-2

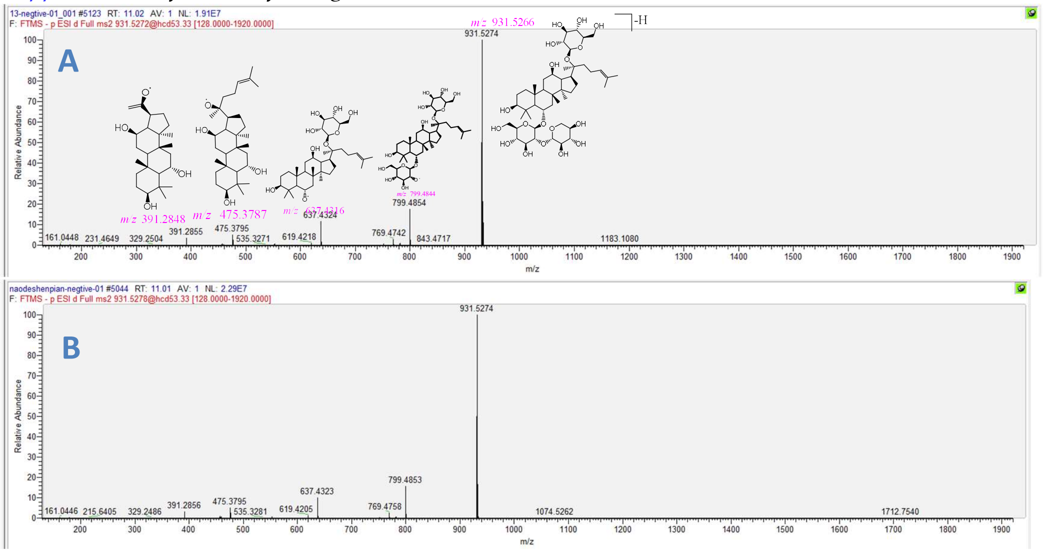

Fig. S36.1 The main results of standard notoginsenoside R1(CAS 80418-24-2, $C_{47}H_{80}O_{18}$)and its corresponding peak in the TIC diagram using UPLC-Q-Orbitrap-MS analysis. (**A**) The MS/MS fragments of standard notoginsenoside R1. (**B**) The MS/MS spectra from chromatographic peak in the Naodeshengpian extract.

Note: The *m/z* values in purple are the calculated ones. The *m/z* calculation was based on the relative atomic masses of C (12.0000), H (1.007825), O (15.994915), and N (14.003074)[1].

Identification: As seen in Fig. S36.1, the R.T. value, molecular ion peak, MS/MS spectra, and characteristic pears were highly similar. Thus, the chromatographic peak in the Naodeshengpian extract was identified as notoginsenoside R1(CAS 80418-24-2, $C_{47}H_{80}O_{18}$).

Suppl. 37 Identification of pratensein CAS 2284-31-3

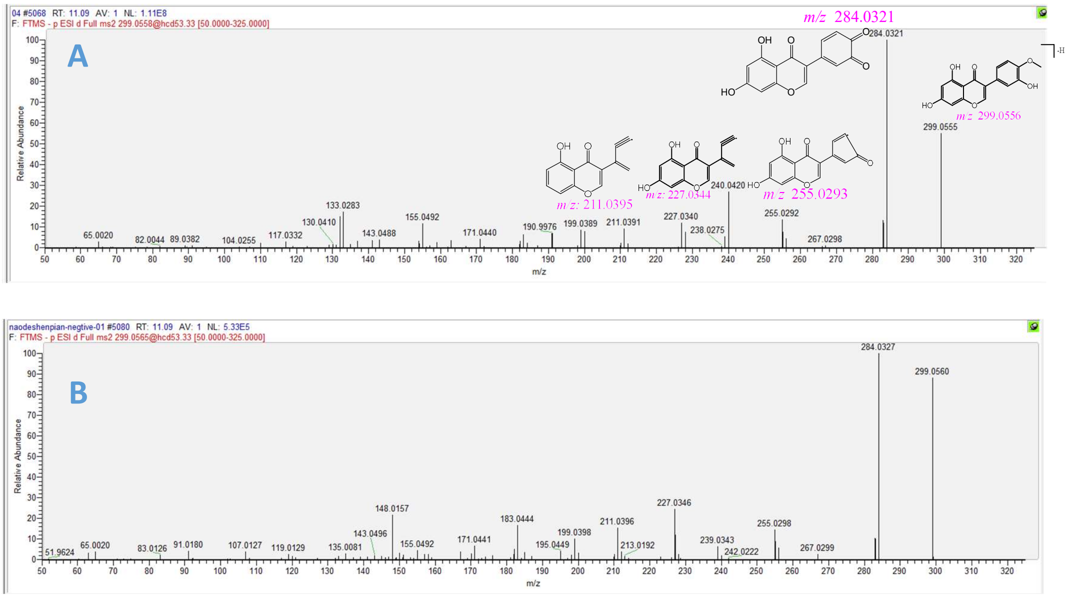

Fig. S37.1 The main results of standard pratensein(CAS 2284-31-3, $C_{16}H_{12}O_6$)and its corresponding peak in the TIC diagram using UPLC-Q-Orbitrap-MS analysis. (**A**) The MS/MS fragments of standard pratensein. (**B**) The MS/MS spectra from chromatographic peak in the Naodeshengpian extract.

Note: The *m/z* values in purple are the calculated ones. The *m/z* calculation was based on the relative atomic masses of C (12.0000), H (1.007825), O (15.994915), and N (14.003074)[1].

Identification: As seen in Fig. S37.1, the R.T. value, molecular ion peak, MS/MS spectra, and characteristic pears were highly similar. Thus, the chromatographic peak in the Naodeshengpian extract was identified as pratensein(CAS 2284-31-3, $C_{16}H_{12}O_6$).

Suppl. 38 Identification of diosmetin CAS 520-34-3

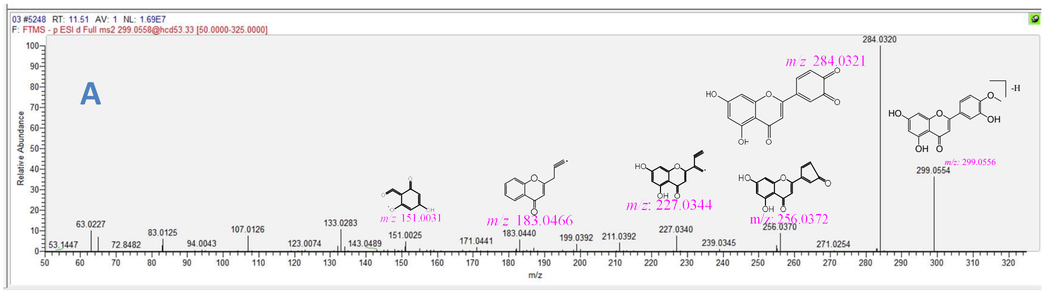

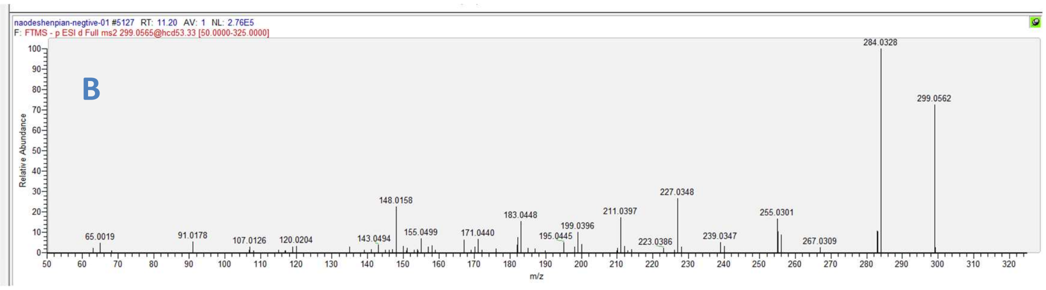

Fig. S38.1 The main results of standard diosmetin(CAS 520-34-3, $C_{16}H_{12}O_6$)and its corresponding peak in the TIC diagram using UPLC-Q-Orbitrap-MS analysis. (**A**) The MS/MS fragments of standard diosmetin. (**B**) The MS/MS spectra from chromatographic peak in the Naodeshengpian extract.

Note: The *m/z* values in purple are the calculated ones. The *m/z* calculation was based on the relative atomic masses of C (12.0000), H (1.007825), O (15.994915), and N (14.003074)[1].

Identification: As seen in Fig. S38.1, the R.T. value, molecular ion peak, MS/MS spectra, and characteristic pears were highly similar. Thus, the chromatographic peak in the Naodeshengpian extract was identified as diosmetin(CAS 520-34-3, $C_{16}H_{12}O_6$).

Suppl. 39 Identification of ginsenoside Rg1 CAS 22427-39-0

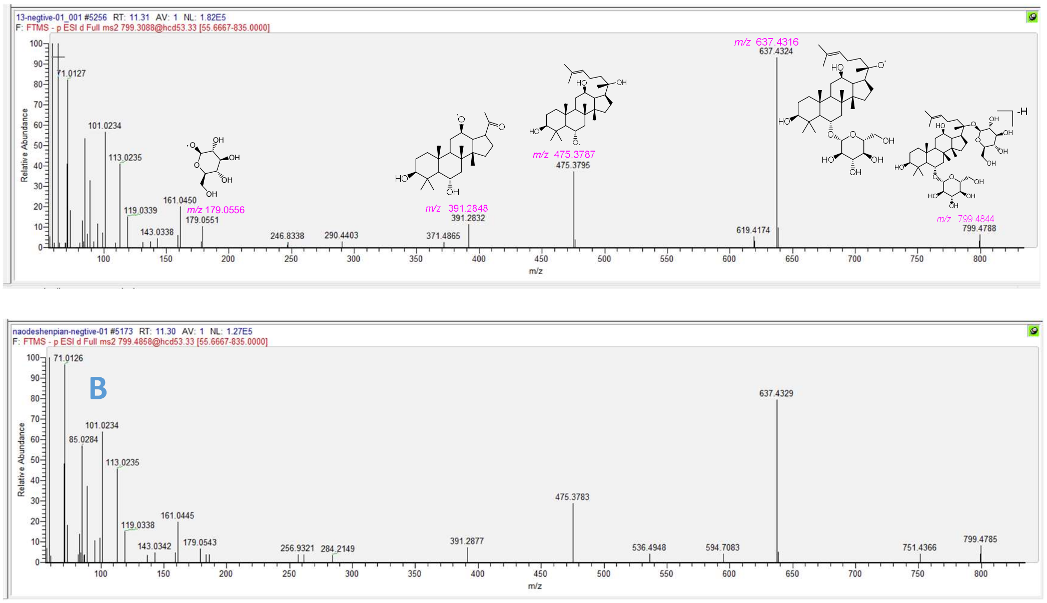

Fig. S39.1 The main results of standard ginsenoside Rg1(CAS 22427-39-0, $C_{42}H_{72}O_{14}$)and its corresponding peak in the TIC diagram using UPLC-Q-Orbitrap-MS analysis. (**A**) The MS/MS fragments of standard ginsenoside Rg1. (**B**) The MS/MS spectra from chromatographic peak in the Naodeshengpian extract.

Note: The *m/z* values in purple are the calculated ones. The *m/z* calculation was based on the relative atomic masses of C (12.0000), H (1.007825), O (15.994915), and N (14.003074)[1].

Identification: As seen in Fig. S39.1, the R.T. value, molecular ion peak, MS/MS spectra, and characteristic pears were highly similar. Thus, the chromatographic peak in the Naodeshengpian extract was identified as ginsenoside Rg1(CAS 22427-39-0, $C_{42}H_{72}O_{14}$).

Suppl. 40 Identification of apigenin CAS 520-36-5

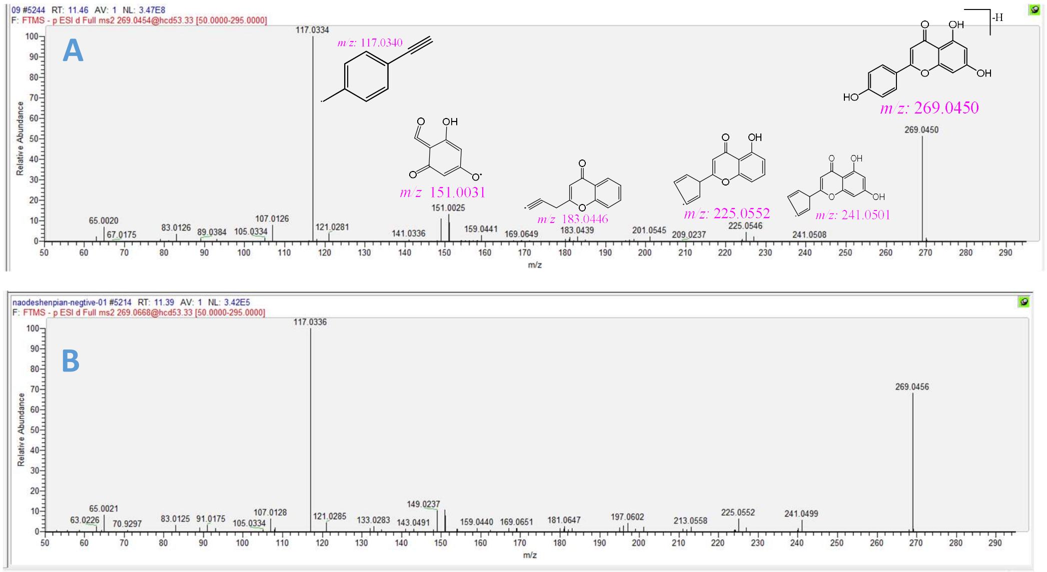

Fig. S40.1 The main results of standard apigenin(CAS 520-36-5, $C_{15}H_{10}O_5$)and its corresponding peak in the TIC diagram using UPLC-Q-Orbitrap-MS analysis. (**A**) The MS/MS fragments of standard apigenin. (**B**) The MS/MS spectra from chromatographic peak in the Naodeshengpian extract.

Note: The *m/z* values in purple are the calculated ones. The *m/z* calculation was based on the relative atomic masses of C (12.0000), H (1.007825), O (15.994915), and N (14.003074)[1].

Identification: As seen in Fig. S40.1, the R.T. value, molecular ion peak, MS/MS spectra, and characteristic pears were highly similar. Thus, the chromatographic peak in the Naodeshengpian extract was identified as apigenin(CAS 520-36-5, $C_{15}H_{10}O_5$).

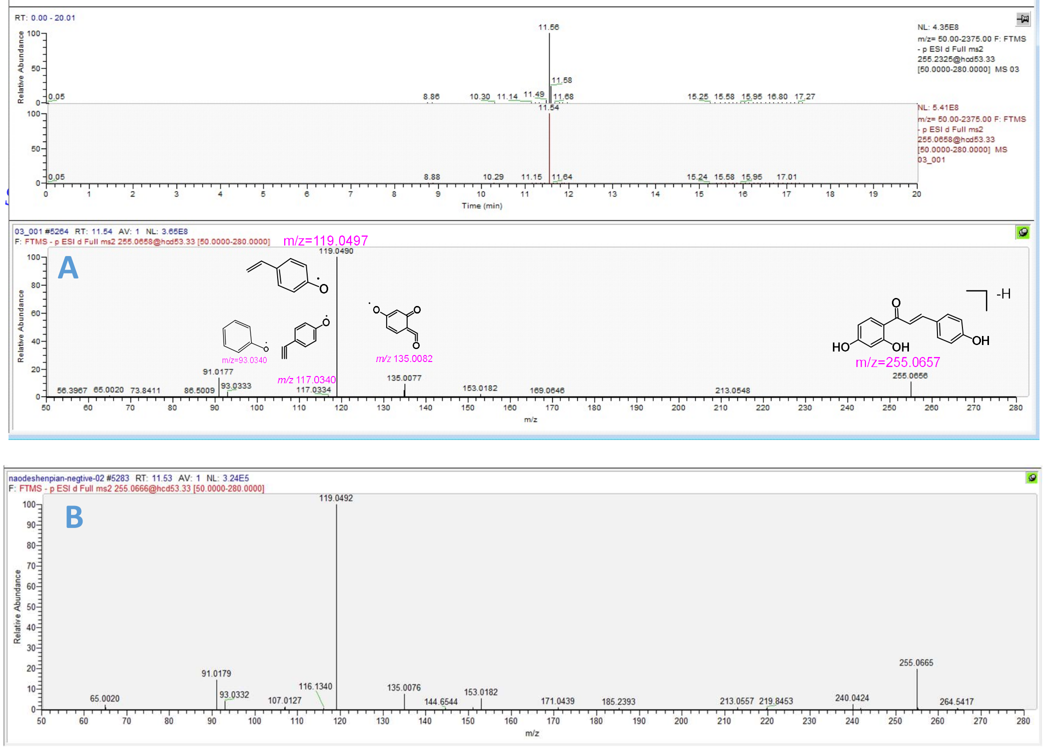

Fig. S41.1 The main results of standard isoliquiritigenin(CAS 961-29-5, $C_{15}H_{12}O_4$)and its corresponding peak in the TIC diagram using UPLC-Q-Orbitrap-MS analysis. (**A**) The MS/MS fragments of standard isoliquiritigenin. (**B**) The MS/MS spectra from chromatographic peak in the Naodeshengpian extract.

Note: The *m/z* values in purple are the calculated ones. The *m/z* calculation was based on the relative atomic masses of C (12.0000), H (1.007825), O (15.994915), and N (14.003074)[1].

Identification: As seen in Fig. S41.1, the R.T. value, molecular ion peak, MS/MS spectra, and characteristic pears were highly similar. Thus, the chromatographic peak in the Naodeshengpian extract was identified as isoliquiritigenin(CAS 961-29-5, $C_{15}H_{12}O_4$).

Suppl. 42 Identification of 7-methoxy-4´-hydroxyisoflavone CAS 486-63-5

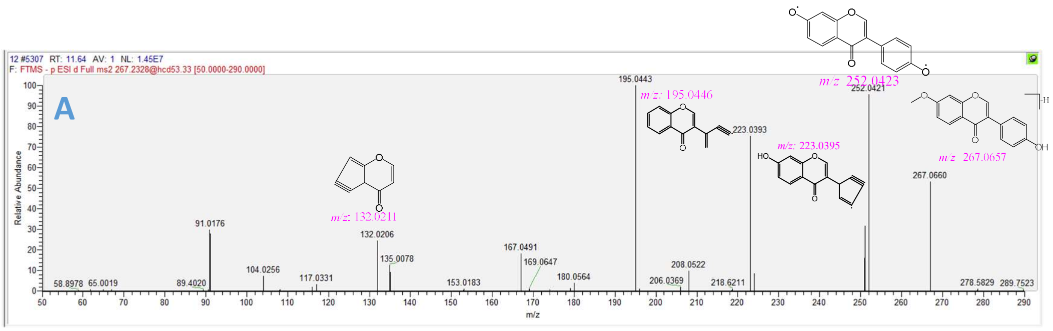

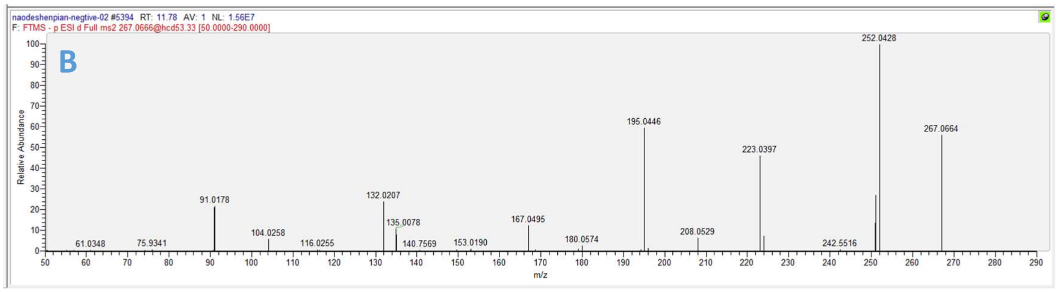

Fig. S42.1 The main results of standard 7-methoxy-4’-hydroxyisoflavone(CAS 961-29-5, $C_{16}H_{12}O_4$)and its corresponding peak in the TIC diagram using UPLC-Q-Orbitrap-MS analysis. (**A**) The MS/MS fragments of standard 7-methoxy-4’-hydroxyisoflavone. (**B**) The MS/MS spectra from chromatographic peak in the Naodeshengpian extract.

Note: The *m/z* values in purple are the calculated ones. The *m/z* calculation was based on the relative atomic masses of C (12.0000), H (1.007825), O (15.994915), and N (14.003074)[1].

Identification: As seen in Fig. S42.1, the R.T. value, molecular ion peak, MS/MS spectra, and characteristic pears were highly similar. Thus, the chromatographic peak in the Naodeshengpian extract was identified as 7-methoxy-4’-hydroxyisoflavone(CAS 961-29-5, $C_{16}H_{12}O_4$).

Suppl. 43 Identification of kaempferol CAS 520-18-3

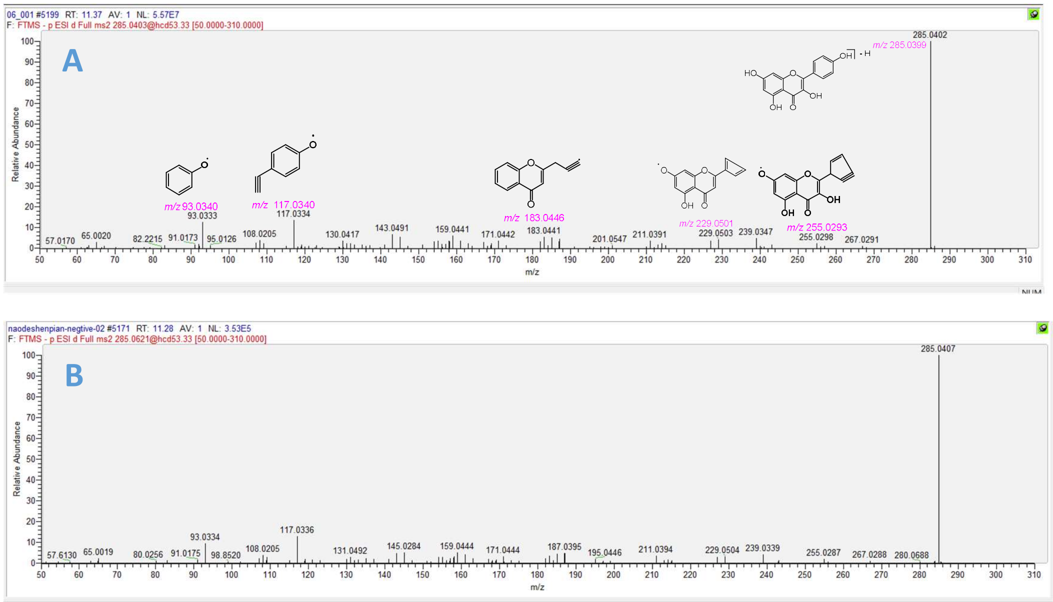

Fig. S43.1 The main results of standard kaempferol(CAS 520-18-3, $C_{15}H_{10}O_6$)and its corresponding peak in the TIC diagram using UPLC-Q-Orbitrap-MS analysis. (**A**) The MS/MS fragments of standard kaempferol. (**B**) The MS/MS spectra from chromatographic peak in the Naodeshengpian extract.

Note: The *m/z* values in purple are the calculated ones. The *m/z* calculation was based on the relative atomic masses of C (12.0000), H (1.007825), O (15.994915), and N (14.003074)[1].

Identification: As seen in Fig. S43.1, the R.T. value, molecular ion peak, MS/MS spectra, and characteristic pears were highly similar. Thus, the chromatographic peak in the Naodeshengpian extract was identified as kaempferol(CAS 520-18-3, $C_{15}H_{10}O_6$).

Suppl. 44 Identification of formononetin CAS 485-72-3

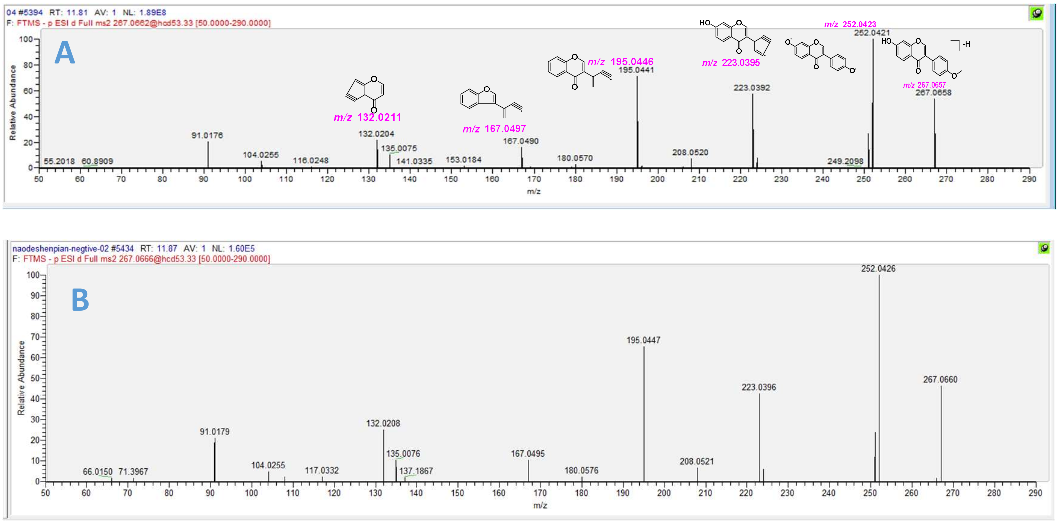

Fig. S44.1 The main results of standard formononetin(CAS 485-72-3, $C_{16}H_{12}O_4$)and its corresponding peak in the TIC diagram using UPLC-Q-Orbitrap-MS analysis. (**A**) The MS/MS fragments of standard formononetin. (**B**) The MS/MS spectra from chromatographic peak in the Naodeshengpian extract.

Note: The *m/z* values in purple are the calculated ones. The *m/z* calculation was based on the relative atomic masses of C (12.0000), H (1.007825), O (15.994915), and N (14.003074)[1].

Identification: As seen in Fig. S44.1, the R.T. value, molecular ion peak, MS/MS spectra, and characteristic pears were highly similar. Thus, the chromatographic peak in the Naodeshengpian extract was identified as formononetin(CAS 485-72-3, $C_{16}H_{12}O_4$).

Suppl. 45 Identification of ginsenoside Rf CAS 52286-58-5

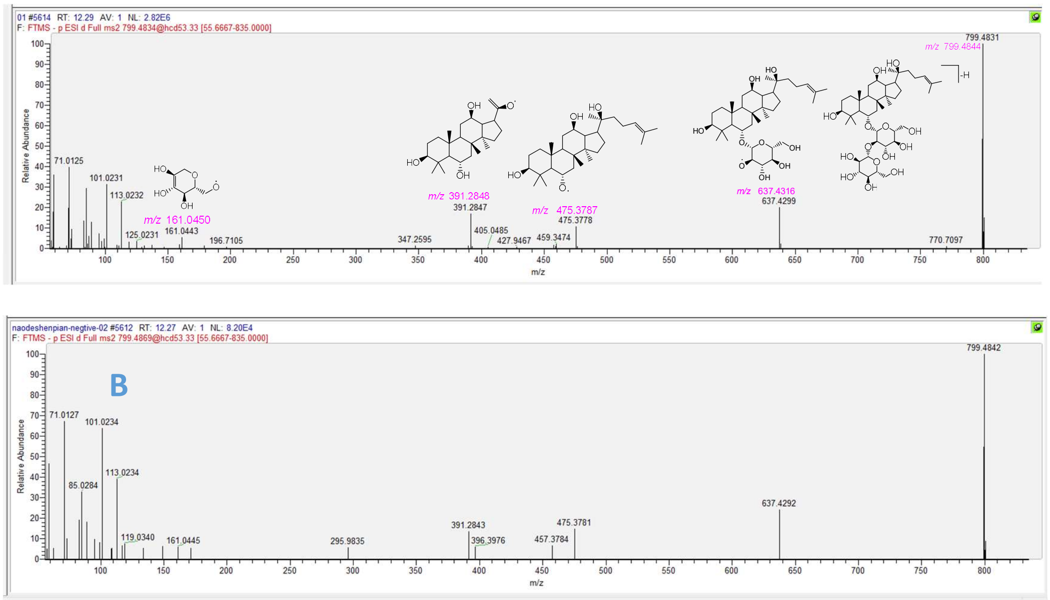

Fig. S45.1 The main results of standard ginsenoside Rf (CAS 52286-58-5, $C_{42}H_{72}O_{14}$)and its corresponding peak in the TIC diagram using UPLC-Q-Orbitrap-MS analysis. (**A**) The MS/MS fragments of standard ginsenoside Rf. (**B**) The MS/MS spectra from chromatographic peak in the Naodeshengpian extract.

Note: The *m/z* values in purple are the calculated ones. The *m/z* calculation was based on the relative atomic masses of C (12.0000), H (1.007825), O (15.994915), and N (14.003074)[1].

Identification: As seen in Fig. S45.1, the R.T. value, molecular ion peak, MS/MS spectra, and characteristic pears were highly similar. Thus, the chromatographic peak in the Naodeshengpian extract was identified as ginsenoside Rf (CAS 52286-58-5, $C_{42}H_{72}O_{14}$).

Suppl. 46 Identification of (r)notoginsenoside R2 CAS 948046-15-9

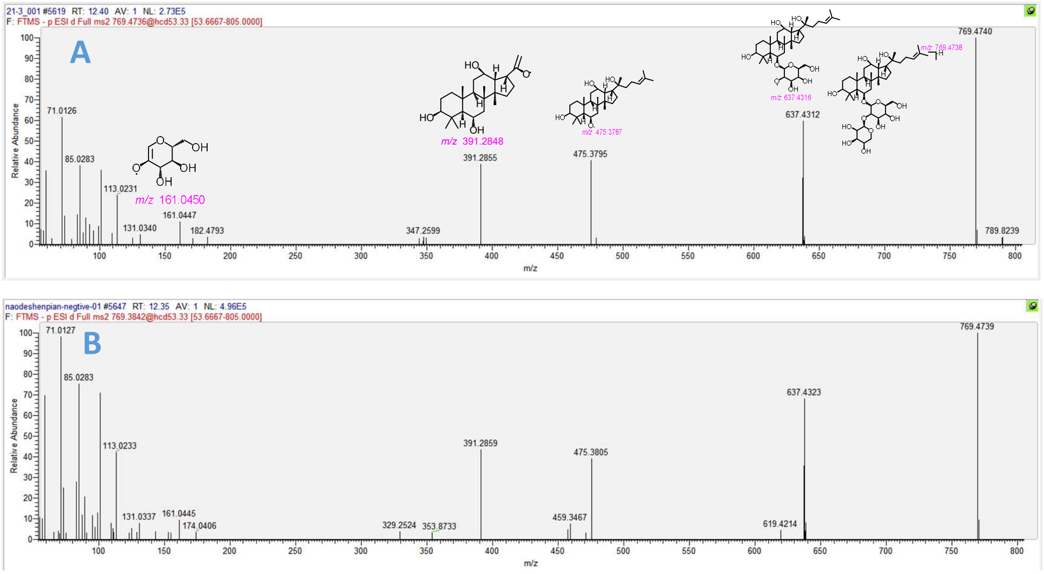

Fig. S46.1 The main results of standard (r)notoginsenoside R2 (CAS 948046-15-9, $C_{41}H_{70}O_{13}$)and its corresponding peak in the TIC diagram using UPLC-Q-Orbitrap-MS analysis. (**A**) The MS/MS fragments of standard (r)notoginsenoside R2. (**B**) The MS/MS spectra from chromatographic peak in the Naodeshengpian extract.

Note: The *m/z* values in purple are the calculated ones. The *m/z* calculation was based on the relative atomic masses of C (12.0000), H (1.007825), O (15.994915), and N (14.003074)[1].

Identification: As seen in Fig. S46.1, the R.T. value, molecular ion peak, MS/MS spectra, and characteristic pears were highly similar. Thus, the chromatographic peak in the Naodeshengpian extract was identified as (r)notoginsenoside R2(CAS 948046-15-9, $C_{41}H_{70}O_{13}$).

Suppl. 47 Identification of prunetin CAS 552-59-0

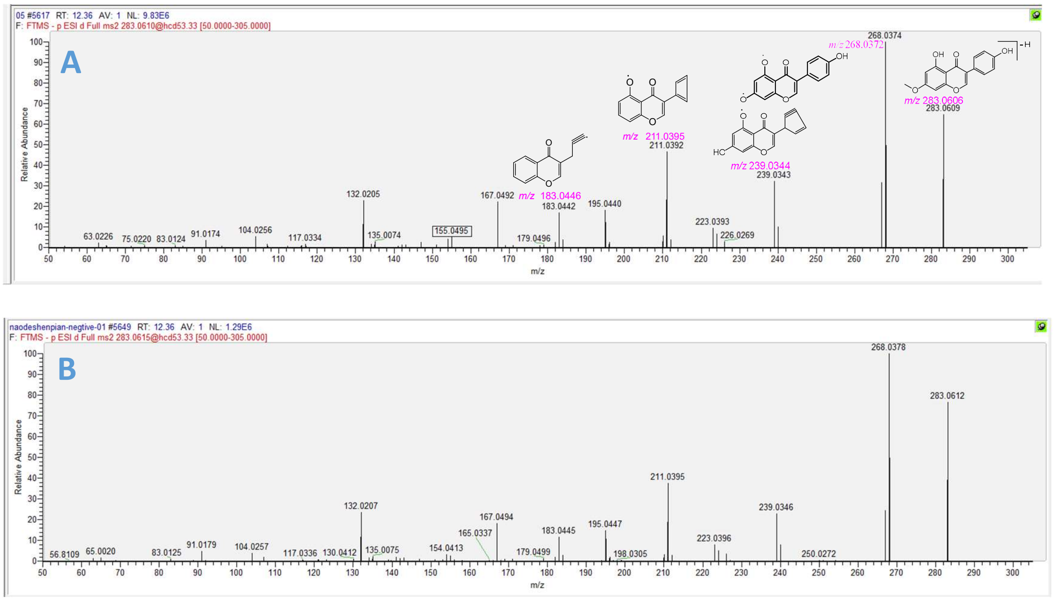

Fig. S47.1 The main results of standard prunetin (CAS 552-59-0, $C_{16}H_{12}O_5$)and its corresponding peak in the TIC diagram using UPLC-Q-Orbitrap-MS analysis. (**A**) The MS/MS fragments of standard prunetin. (**B**) The MS/MS spectra from chromatographic peak in the Naodeshengpian extract.

Note: The *m/z* values in purple are the calculated ones. The *m/z* calculation was based on the relative atomic masses of C (12.0000), H (1.007825), O (15.994915), and N (14.003074)[1].

Identification: As seen in Fig. S47.1, the R.T. value, molecular ion peak, MS/MS spectra, and characteristic pears were highly similar. Thus, the chromatographic peak in the Naodeshengpian extract was identified as prunetin(CAS 552-59-0, $C_{16}H_{12}O_5$).

Suppl. 48 Identification of ginsenoside Rg2 CAS 52286-74-5

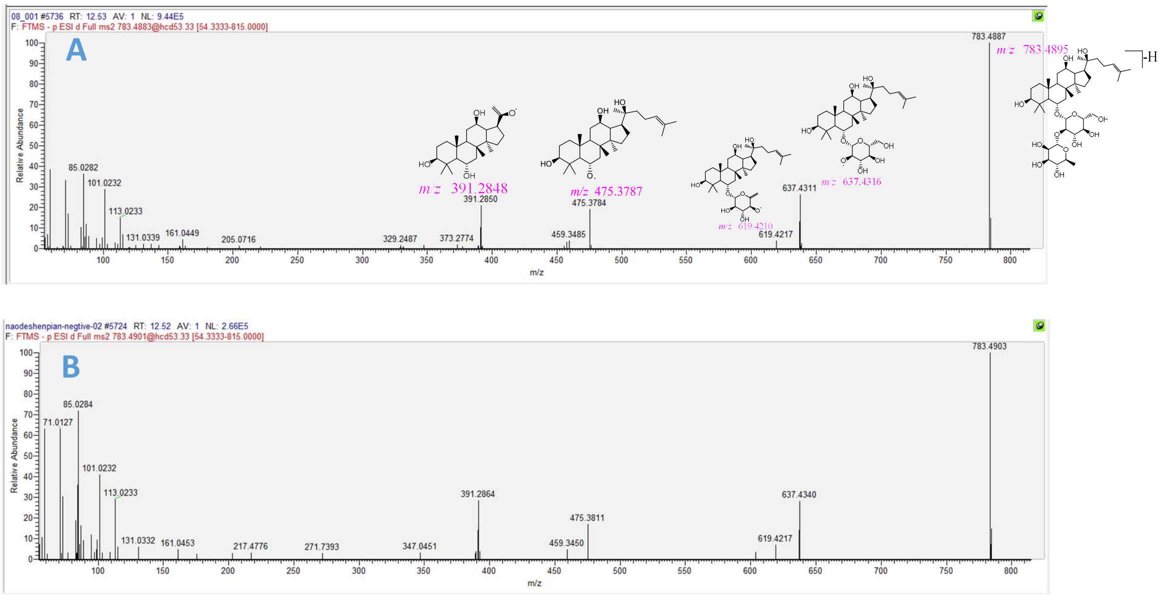

Fig. S48.1 The main results of standard ginsenoside Rg2 (CAS 52286-74-5, $C_{42}H_{72}O_{13}$)and its corresponding peak in the TIC diagram using UPLC-Q-Orbitrap-MS analysis. (**A**) The MS/MS fragments of standard ginsenoside Rg2. (**B**) The MS/MS spectra from chromatographic peak in the Naodeshengpian extract.

Note: The *m/z* values in purple are the calculated ones. The *m/z* calculation was based on the relative atomic masses of C (12.0000), H (1.007825), O (15.994915), and N (14.003074)[1].

Identification: As seen in Fig. S48.1, the R.T. value, molecular ion peak, MS/MS spectra, and characteristic pears were highly similar. Thus, the chromatographic peak in the Naodeshengpian extract was identified as ginsenoside Rg2(CAS 52286-74-5, $C_{42}H_{72}O_{13}$).

Suppl. 49 Identification of 20S-ginsenoside Rh1 CAS 63223-86-9

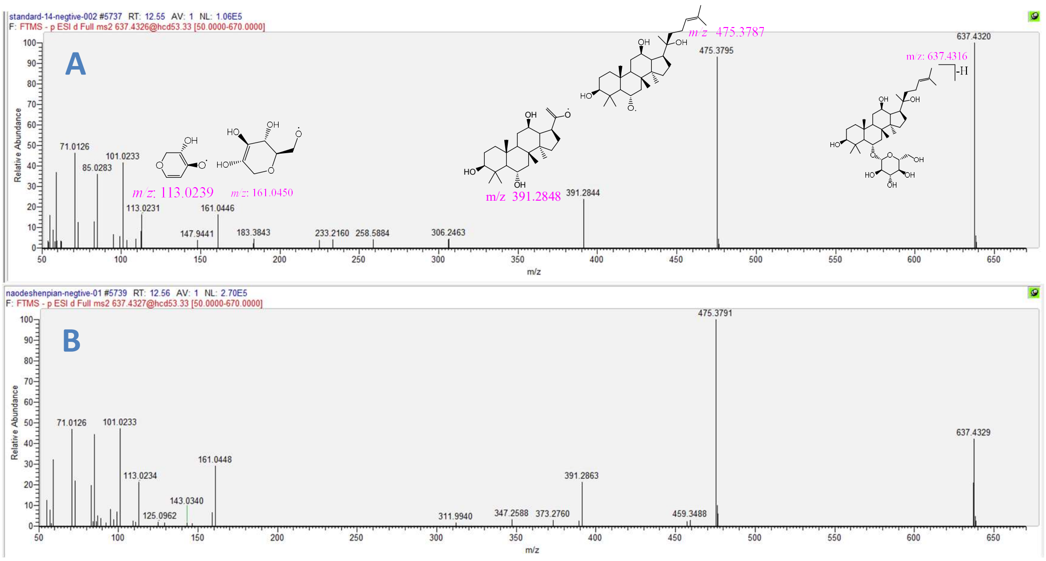

Fig. S49.1 The main results of standard 20S-ginsenoside Rh1 (CAS 63223-86-9, $C_{36}H_{62}O_9$)and its corresponding peak in the TIC diagram using UPLC-Q-Orbitrap-MS analysis. (**A**) The MS/MS fragments of standard 20S-ginsenoside Rh1. (**B**) The MS/MS spectra from chromatographic peak in the Naodeshengpian extract.

Note: The *m/z* values in purple are the calculated ones. The *m/z* calculation was based on the relative atomic masses of C (12.0000), H (1.007825), O (15.994915), and N (14.003074)[1].

Identification: As seen in Fig. S49.1, the R.T. value, molecular ion peak, MS/MS spectra, and characteristic pears were highly similar. Thus, the chromatographic peak in the Naodeshengpian extract was identified as 20S-ginsenoside Rh1(CAS 63223-86-9, $C_{36}H_{62}O_9$).

Suppl. 50 Identification of ginsenoside Rb1   CAS 41753-43-9

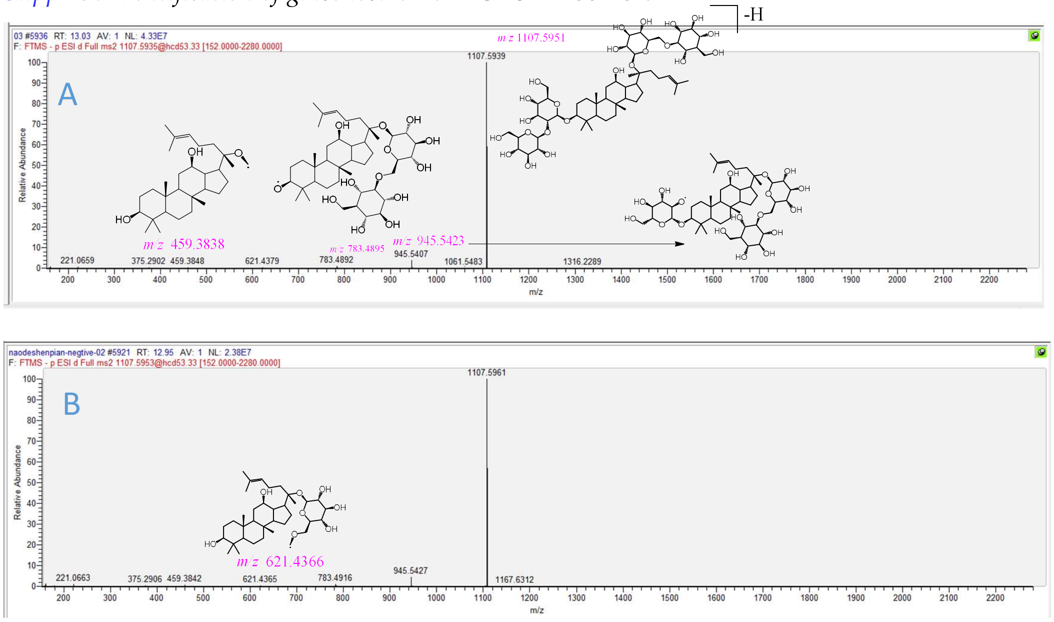

Fig. S50.1 The main results of standard ginsenoside Rb1 (CAS 41753-43-9, $C_{54}H_{92}O_{63}$)and its corresponding peak in the TIC diagram using UPLC-Q-Orbitrap-MS analysis. (**A**) The MS/MS fragments of standard ginsenoside Rb1. (**B**) The MS/MS spectra from chromatographic peak in the Naodeshengpian extract.

Note: The *m/z* values in purple are the calculated ones. The *m/z* calculation was based on the relative atomic masses of C (12.0000), H (1.007825), O (15.994915), and N (14.003074)[1].

Identification: As seen in Fig. S50.1, the R.T. value, molecular ion peak, MS/MS spectra, and characteristic pears were highly similar. Thus, the chromatographic peak in the Naodeshengpian extract was identified as ginsenoside Rb1(CAS 41753-43-9, $C_{54}H_{92}O_{63}$).

Suppl. 51 Identification of 8-prenyldaidzein CAS 135384-00-8

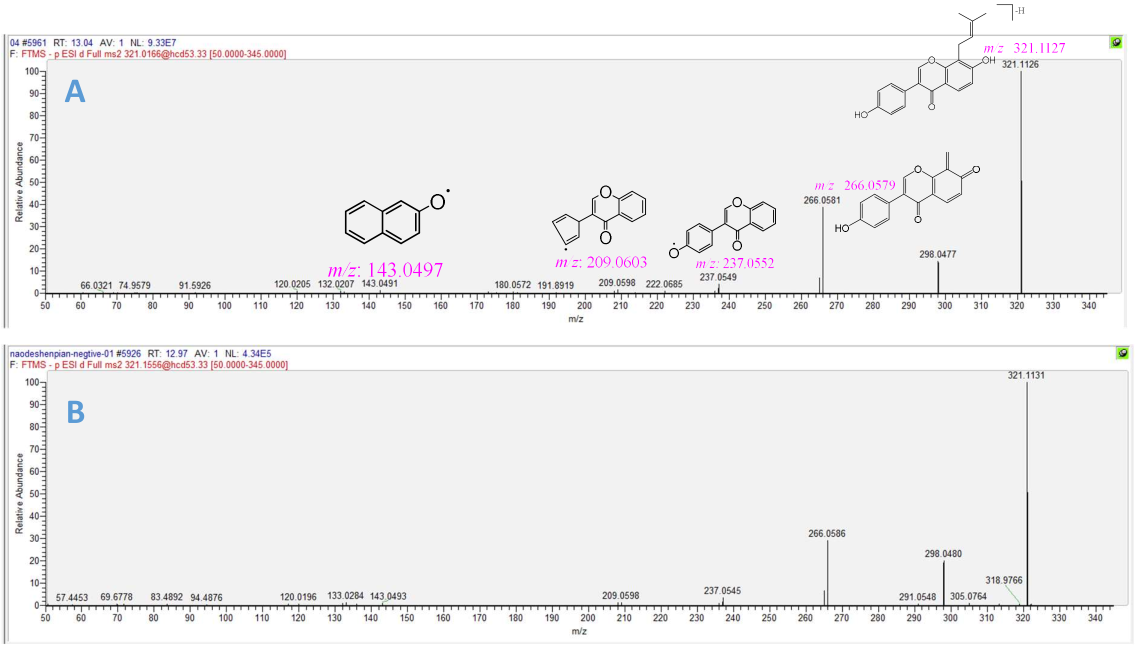

Fig. S51.1 The main results of standard 8-prenyldaidzein (CAS 135384-00-8, $C_{20}H_{18}O_4$)and its corresponding peak in the TIC diagram using UPLC-Q-Orbitrap-MS analysis. (**A**) The MS/MS fragments of standard 8-prenyldaidzein. (**B**) The MS/MS spectra from chromatographic peak in the Naodeshengpian extract.

Note: The *m/z* values in purple are the calculated ones. The *m/z* calculation was based on the relative atomic masses of C (12.0000), H (1.007825), O (15.994915), and N (14.003074)[1].

Identification: As seen in Fig. S51.1, the R.T. value, molecular ion peak, MS/MS spectra, and characteristic pears were highly similar. Thus, the chromatographic peak in the Naodeshengpian extract was identified as 8-prenyldaidzein(CAS 135384-00-8, $C_{20}H_{18}O_4$).

Suppl. 52 Identification of ginsenoside Rd CAS 52705-93-8

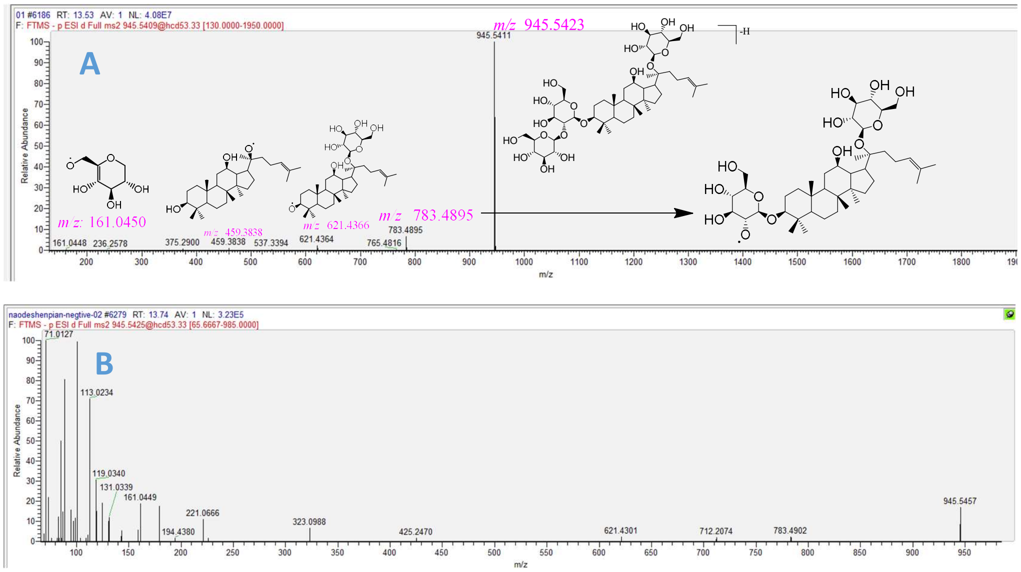

Fig. S52.1 The main results of standard ginsenoside Rd (CAS 52705-93-8, $C_{48}H_{82}O_{18}$)and its corresponding peak in the TIC diagram using UPLC-Q-Orbitrap-MS analysis. (**A**) The MS/MS fragments of standard ginsenoside Rd. (**B**) The MS/MS spectra from chromatographic peak in the Naodeshengpian extract.

Note: The *m/z* values in purple are the calculated ones. The *m/z* calculation was based on the relative atomic masses of C (12.0000), H (1.007825), O (15.994915), and N (14.003074)[1].

Identification: As seen in Fig. S52.1, the R.T. value, molecular ion peak, MS/MS spectra, and characteristic pears were highly similar. Thus, the chromatographic peak in the Naodeshengpian extract was identified as ginsenoside Rd(CAS 52705-93-8, $C_{48}H_{82}O_{18}$).

Suppl. 53 Identification of ginsenoside Rg3 CAS 14197-60-5

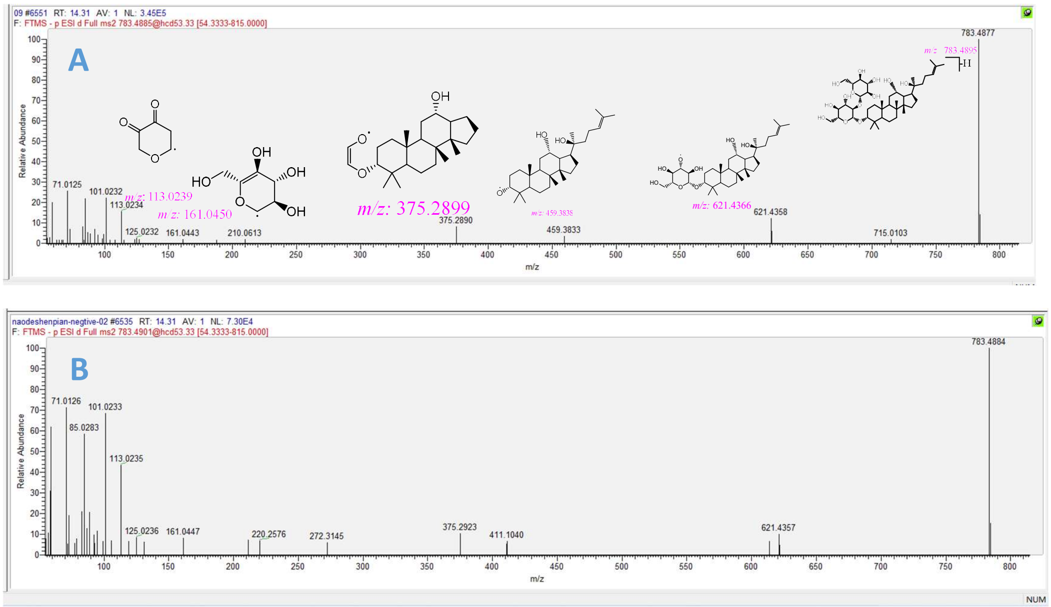

Fig. S53.1 The main results of standard ginsenoside Rg3 (CAS 14197-60-5, $C_{42}H_{72}O_{13}$)and its corresponding peak in the TIC diagram using UPLC-Q-Orbitrap-MS analysis. (**A**) The MS/MS fragments of standard ginsenoside Rg3. (**B**) The MS/MS spectra from chromatographic peak in the Naodeshengpian extract.

Note: The *m/z* values in purple are the calculated ones. The *m/z* calculation was based on the relative atomic masses of C (12.0000), H (1.007825), O (15.994915), and N (14.003074)[1].

Identification: As seen in Fig. S53.1, the R.T. value, molecular ion peak, MS/MS spectra, and characteristic pears were highly similar. Thus, the chromatographic peak in the Naodeshengpian extract was identified as ginsenoside Rg3(CAS 14197-60-5, $C_{42}H_{72}O_{13}$).

Suppl. 54 Identification of ethyl stearate CAS 111-61-5

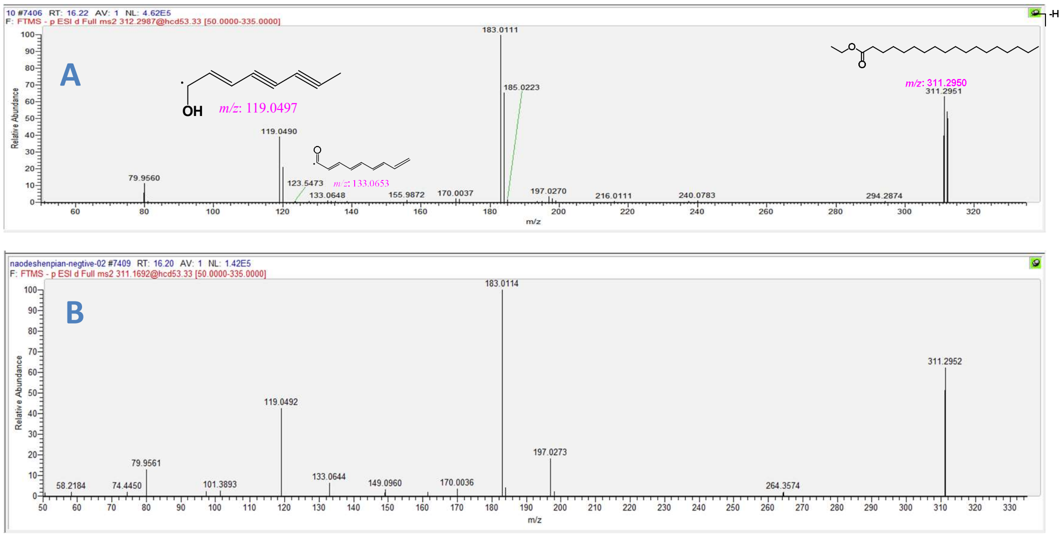

Fig. S54.1 The main results of standard ethyl stearate(CAS 111-61-5, $C_{20}H_{40}O_2$)and its corresponding peak in the TIC diagram using UPLC-Q-Orbitrap-MS analysis. (**A**) The MS/MS fragments of standard ethyl stearate. (**B**) The MS/MS spectra from chromatographic peak in the Naodeshengpian extract.

Note: The *m/z* values in purple are the calculated ones. The *m/z* calculation was based on the relative atomic masses of C (12.0000), H (1.007825), O (15.994915), and N (14.003074)[1].

Identification: As seen in Fig. S54.1, the R.T. value, molecular ion peak, MS/MS spectra, and characteristic pears were highly similar. Thus, the chromatographic peak in the Naodeshengpian extract was identified as ethyl stearate(CAS 111-61-5, $C_{20}H_{40}O_2$).

Suppl. 55 Identification of matrine CAS 519-02-8

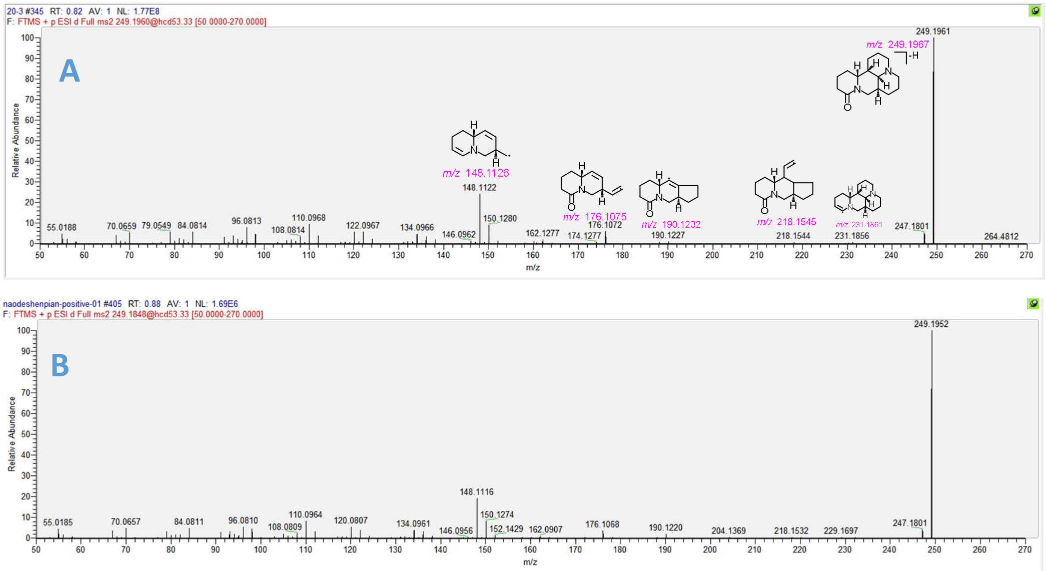

Fig. S55.1 The main results of standard matrine(CAS 519-02-8, $C_{15}H_{24}N_2O$)and its corresponding peak in the TIC diagram using UPLC-Q-Orbitrap-MS analysis. (**A**) The MS/MS fragments of standard matrine. (**B**) The MS/MS spectra from chromatographic peak in the Naodeshengpian extract.

Note: The *m/z* values in purple are the calculated ones. The *m/z* calculation was based on the relative atomic masses of C (12.0000), H (1.007825), O (15.994915), and N (14.003074)[1].

Identification: As seen in Fig. S55.1, the R.T. value, molecular ion peak, MS/MS spectra, and characteristic pears were highly similar. Thus, the chromatographic peak in the Naodeshengpian extract was identified as matrine(CAS 519-02-8, $C_{15}H_{24}N_2O$).

Suppl. 56 Identification of 5-hydroxymethylfurfural CAS 67-47-0

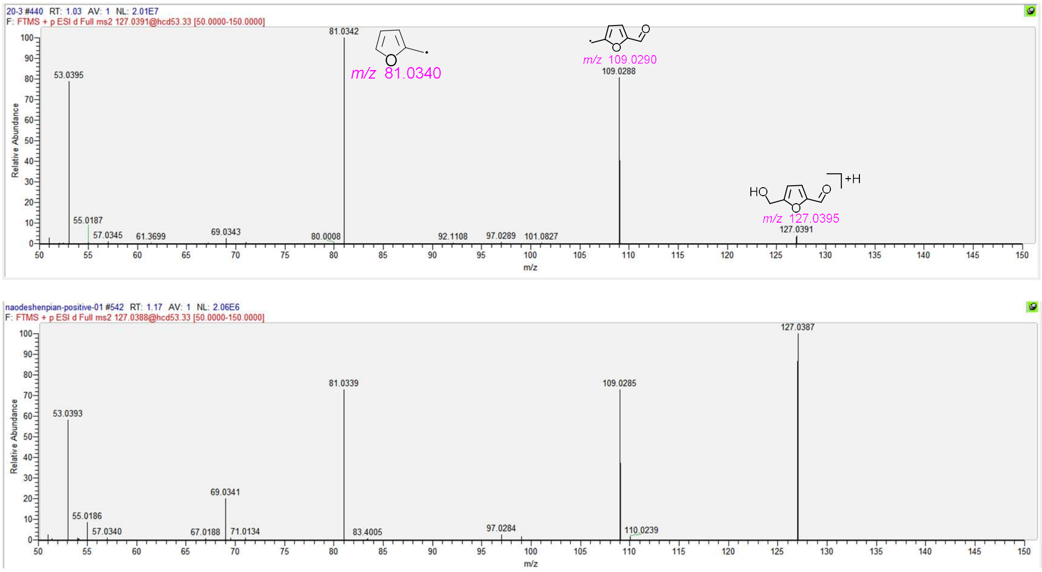

Fig. S56.1 The main results of standard 5-hydroxymethylfurfural(CAS 67-47-0, $C_6H_6O_3$)and its corresponding peak in the TIC diagram using UPLC-Q-Orbitrap-MS analysis. (**A**) The MS/MS fragments of standard 5-hydroxymethylfurfural. (**B**) The MS/MS spectra from chromatographic peak in the Naodeshengpian extract.

Note: The *m/z* values in purple are the calculated ones. The *m/z* calculation was based on the relative atomic masses of C (12.0000), H (1.007825), O (15.994915), and N (14.003074)[1].

Identification: As seen in Fig. S56.1, the R.T. value, molecular ion peak, MS/MS spectra, and characteristic pears were highly similar. Thus, the chromatographic peak in the Naodeshengpian extract was identified as5-hydroxymethylfurfural(CAS 67-47-0, $C_6H_6O_3$).

Suppl. 57 Identification of caffeine CAS 58-08-2

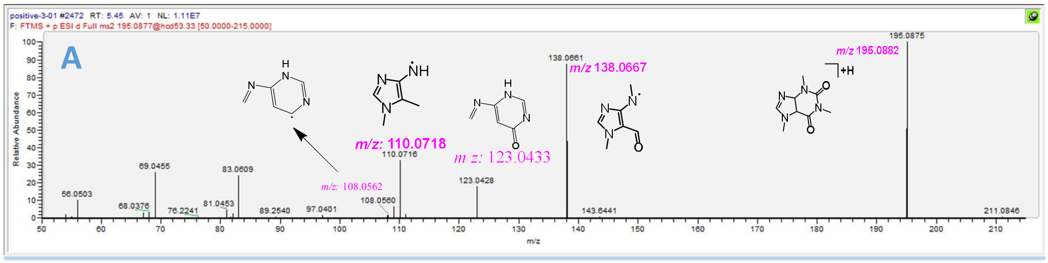

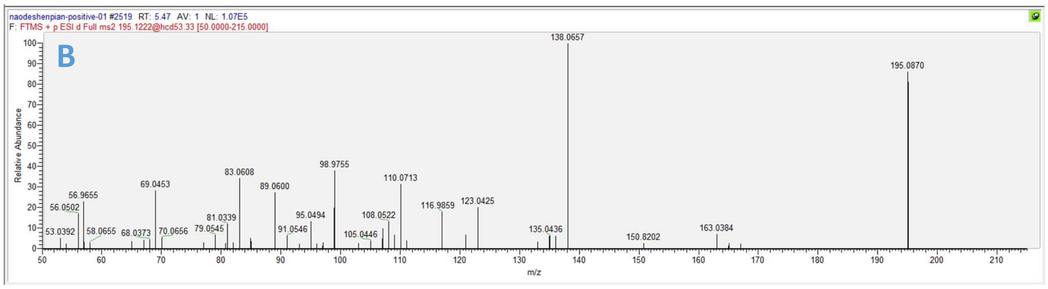

Fig. S57.1 The main results of standard caffeine(CAS 58-08-2, $C_8H_{10}N_4O_2$)and its corresponding peak in the TIC diagram using UPLC-Q-Orbitrap-MS analysis. (**A**) The MS/MS fragments of standard caffeine. (**B**) The MS/MS spectra from chromatographic peak in the Naodeshengpian extract.

Note: The *m/z* values in purple are the calculated ones. The *m/z* calculation was based on the relative atomic masses of C (12.0000), H (1.007825), O (15.994915), and N (14.003074)[1].

Identification: As seen in Fig. S57.1, the R.T. value, molecular ion peak, MS/MS spectra, and characteristic pears were highly similar. Thus, the chromatographic peak in the Naodeshengpian extract was identified as caffeine(CAS 58-08-2, $C_8H_{10}N_4O_2$).

Suppl. 58 Identification of 1,5-dicaffeoylquinic acid CAS 30964-13-7

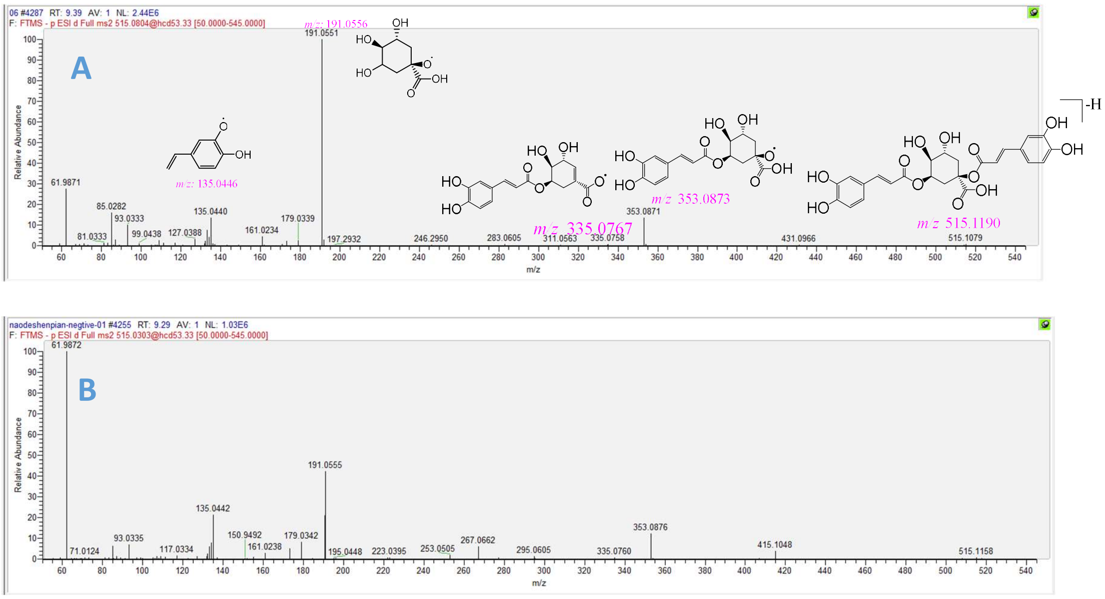

Fig. S58.1 The main results of standard 1,5-dicaffeoylquinic acid(CAS 30964-13-7, $C_{25}H_{24}O_{12}$)and its corresponding peak in the TIC diagram using UPLC-Q-Orbitrap-MS analysis. (**A**) The MS/MS fragments of standard 1,5-dicaffeoylquinic acid. (**B**) The MS/MS spectra from chromatographic peak in the Naodeshengpian extract.

Note: The *m/z* values in purple are the calculated ones. The *m/z* calculation was based on the relative atomic masses of C (12.0000), H (1.007825), O (15.994915), and N (14.003074)[1].

Identification: As seen in Fig. S58.1, the R.T. value, molecular ion peak, MS/MS spectra, and characteristic pears were highly similar. Thus, the chromatographic peak in the Naodeshengpian extract was identified as 1,5-dicaffeoylquinic acid(CAS 30964-13-7, $C_{25}H_{24}O_{12}$).

Suppl. 59 Identification of scoparone CAS 120-08-1

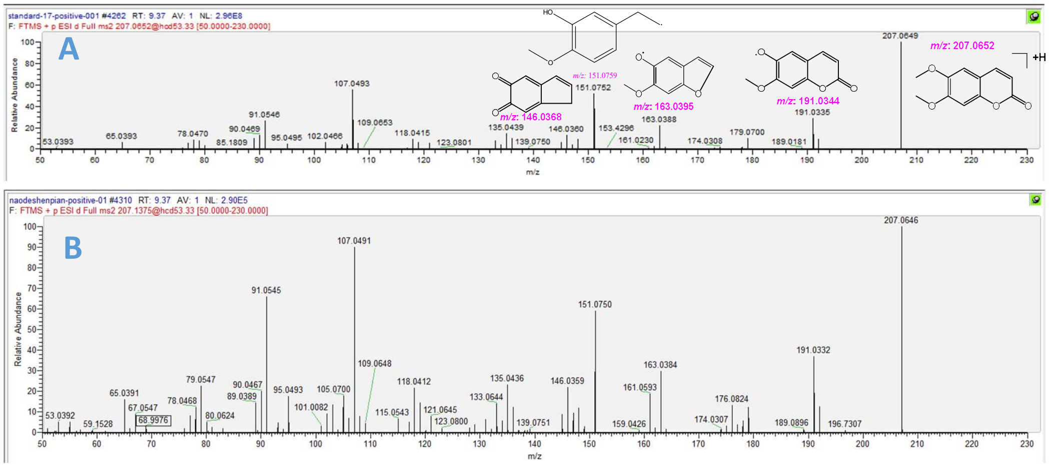

Fig. S59.1 The main results of standard scoparone(CAS 120-08-1, $C_{11}H_{10}O_4$)and its corresponding peak in the TIC diagram using UPLC-Q-Orbitrap-MS analysis. (**A**) The MS/MS fragments of standard scoparone. (**B**) The MS/MS spectra from chromatographic peak in the Naodeshengpian extract.

Note：The *m/z* values in purple are the calculated ones. The *m/z* calculation was based on the relative atomic masses of C (12.0000), H (1.007825), O (15.994915), and N (14.003074)[1].

Identification: As seen in Fig. S59.1, the R.T. value, molecular ion peak, MS/MS spectra, and characteristic pears were highly similar. Thus, the chromatographic peak in the Naodeshengpian extract was identified as scoparone(CAS 120-08-1, $C_{11}H_{10}O_4$).

Suppl. 60 Identification of senkyunolide A CAS 63038-10-8

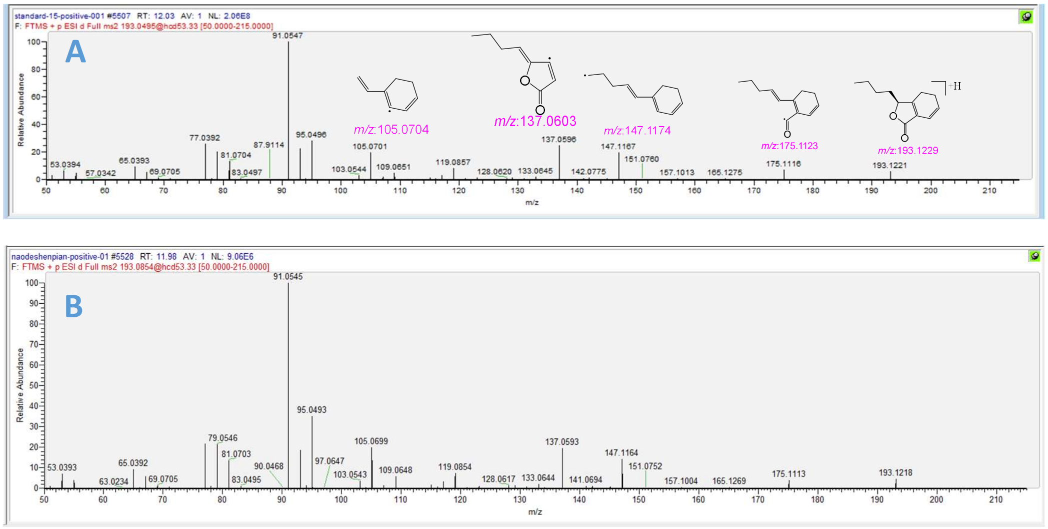

Fig. S60.1 The main results of standard senkyunolide A(CAS 63038-10-8, $C_{12}H_{16}O_2$)and its corresponding peak in the TIC diagram using UPLC-Q-Orbitrap-MS analysis. (**A**) The MS/MS fragments of standard senkyunolide A. (**B**) The MS/MS spectra from chromatographic peak in the Naodeshengpian extract.

Note: The *m/z* values in purple are the calculated ones. The *m/z* calculation was based on the relative atomic masses of C (12.0000), H (1.007825), O (15.994915), and N (14.003074)[1].

Identification: As seen in Fig. S60.1, the R.T. value, molecular ion peak, MS/MS spectra, and characteristic pears were highly similar. Thus, the chromatographic peak in the Naodeshengpian extract was identified as senkyunolide A(CAS 63038-10-8, $C_{12}H_{16}O_2$).

Suppl. 61 Identification of ligustilide CAS 81944-09-4

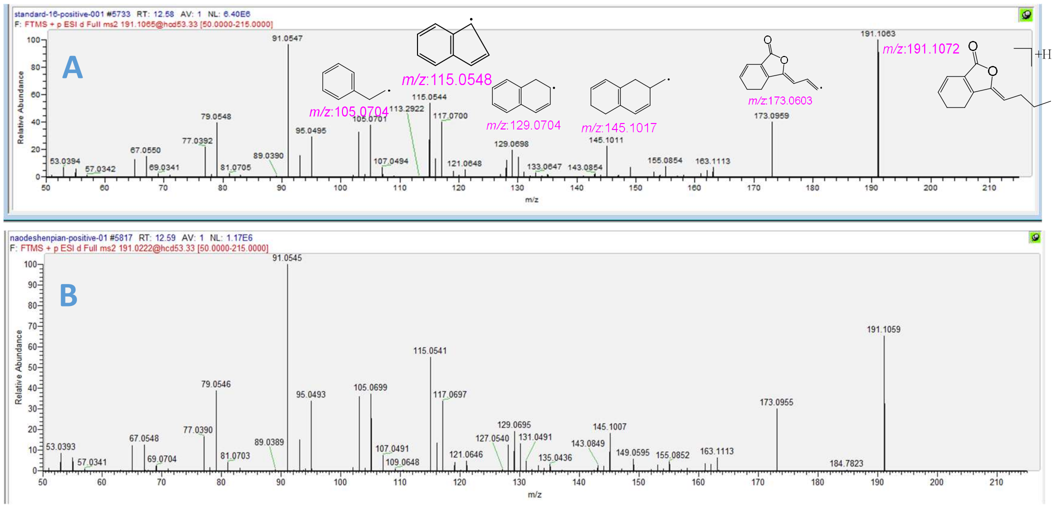

Fig. S61.1 The main results of standard ligustilide(CAS 81944-09-4, $C_{12}H_{14}O_2$)and its corresponding peak in the TIC diagram using UPLC-Q-Orbitrap-MS analysis. (**A**) The MS/MS fragments of standard ligustilide. (**B**) The MS/MS spectra from chromatographic peak in the Naodeshengpian extract.

Note: The *m/z* values in purple are the calculated ones. The *m/z* calculation was based on the relative atomic masses of C (12.0000), H (1.007825), O (15.994915), and N (14.003074)[1].

Identification: As seen in Fig. S61.1, the R.T. value, molecular ion peak, MS/MS spectra, and characteristic pears were highly similar. Thus, the chromatographic peak in the Naodeshengpian extract was identified as ligustilide(CAS 81944-09-4, $C_{12}H_{14}O_2$).

Suppl. 62 Identification of 3,3′,4′,5,6,7,8-heptamethoxyflavone CAS 1178-24-1

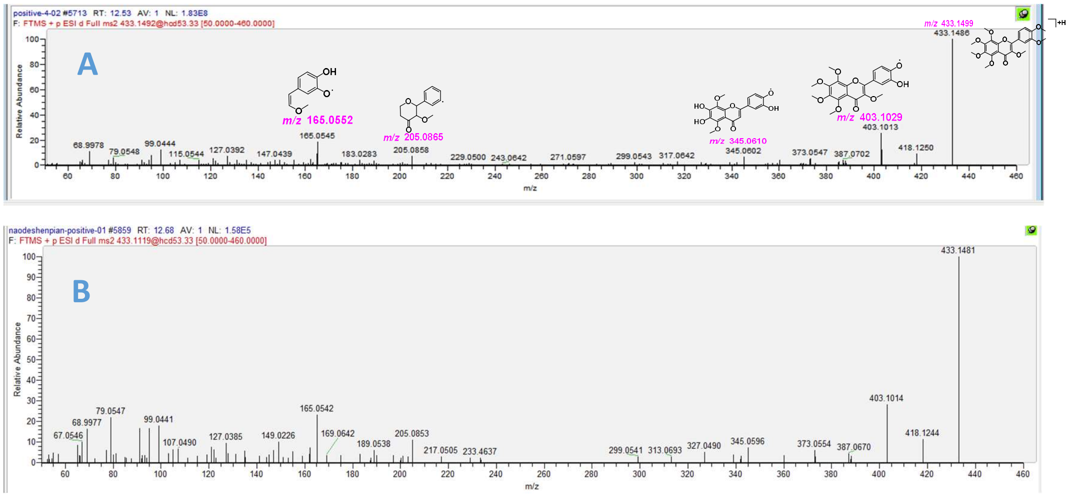

Fig. S62.1 The main results of standard 3,3',4',5,6,7,8-heptamethoxyflavone(CAS 1178-24-1, $C_{22}H_{24}O_9$)and its corresponding peak in the TIC diagram using UPLC-Q-Orbitrap-MS analysis. (**A**) The MS/MS fragments of standard 3,3',4',5,6,7,8-heptamethoxyflavone. (**B**) The MS/MS spectra from chromatographic peak in the Naodeshengpian extract.

Note: The *m/z* values in purple are the calculated ones. The *m/z* calculation was based on the relative atomic masses of C (12.0000), H (1.007825), O (15.994915), and N (14.003074)[1].

Identification: As seen in Fig. S62.1, the R.T. value, molecular ion peak, MS/MS spectra, and characteristic pears were highly similar. Thus, the chromatographic peak in the Naodeshengpian extract was identified as 3,3',4',5,6,7,8-heptamethoxyflavone(CAS 1178-24-1, $C_{22}H_{24}O_9$).

Suppl. 63 Identification of tangeretin CAS 481-53-8

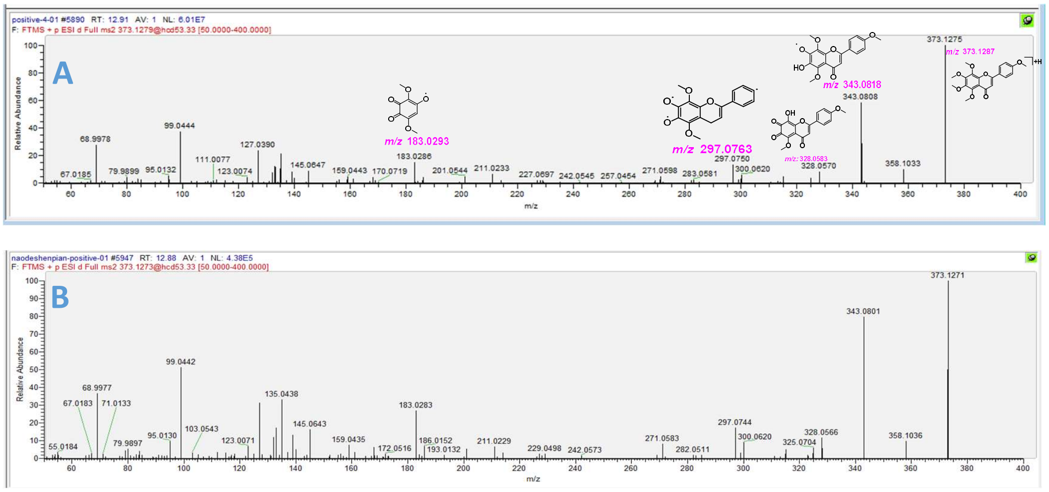

Fig. S63.1 The main results of standard tangeretin(CAS 481-53-8, $C_{20}H_{20}O_7$)and its corresponding peak in the TIC diagram using UPLC-Q-Orbitrap-MS analysis. (**A**) The MS/MS fragments of standard tangeretin. (**B**) The MS/MS spectra from chromatographic peak in the Naodeshengpian extract.

Note: The *m/z* values in purple are the calculated ones. The *m/z* calculation was based on the relative atomic masses of C (12.0000), H (1.007825), O (15.994915), and N (14.003074)[1].

Identification: As seen in Fig. S63.1, the R.T. value, molecular ion peak, MS/MS spectra, and characteristic pears were highly similar. Thus, the chromatographic peak in the Naodeshengpian extract was identified as tangeretin(CAS 481-53-8, $C_{20}H_{20}O_7$).

Suppl. 64 Identification of 5-hydroxyflavone CAS 491-78-1

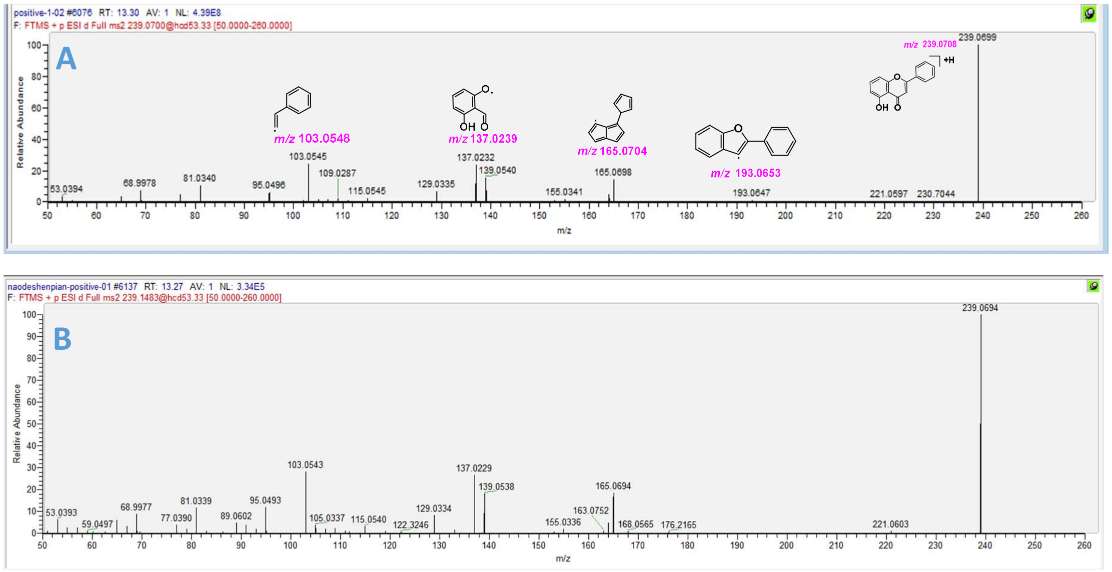

Fig. S64.1 The main results of standard 5-hydroxyflavone(CAS 491-78-1, $C_{15}H_{10}O_3$)and its corresponding peak in the TIC diagram using UPLC-Q-Orbitrap-MS analysis. (**A**) The MS/MS fragments of standard 5-hydroxyflavone. (**B**) The MS/MS spectra from chromatographic peak in the Naodeshengpian extract.

Note: The *m/z* values in purple are the calculated ones. The *m/z* calculation was based on the relative atomic masses of C (12.0000), H (1.007825), O (15.994915), and N (14.003074)[1].

Identification: As seen in Fig. S64.1, the R.T. value, molecular ion peak, MS/MS spectra, and characteristic pears were highly similar. Thus, the chromatographic peak in the Naodeshengpian extract was identified as 5-hydroxyflavone(CAS 491-78-1, $C_{15}H_{10}O_3$).

Suppl. 65 Identification of levistilide A CAS 88182-33-6

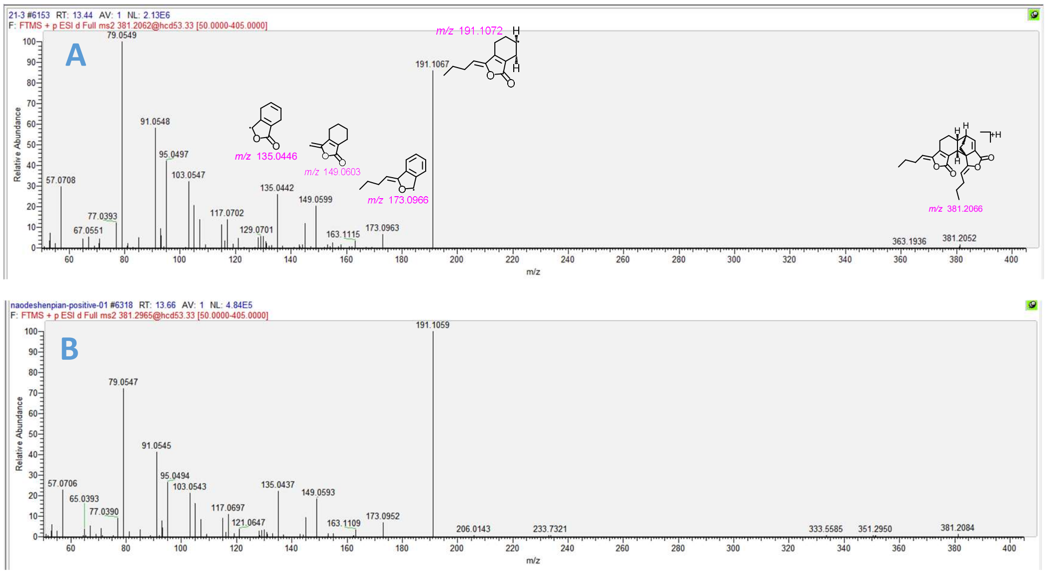

Fig. S65.1 The main results of standard levistilide A(CAS 88182-33-6, $C_{24}H_{28}O_4$)and its corresponding peak in the TIC diagram using UPLC-Q-Orbitrap-MS analysis. (**A**) The MS/MS fragments of standard levistilide A. (**B**) The MS/MS spectra from chromatographic peak in the Naodeshengpian extract.

Note: The *m/z* values in purple are the calculated ones. The *m/z* calculation was based on the relative atomic masses of C (12.0000), H (1.007825), O (15.994915), and N (14.003074)[1].

Identification: As seen in Fig. S65.1, the R.T. value, molecular ion peak, MS/MS spectra, and characteristic pears were highly similar. Thus, the chromatographic peak in the Naodeshengpian extract was identified as levistilide A(CAS 88182-33-6, $C_{24}H_{28}O_4$).

Suppl. 66 Identification of diosgenin CAS 512-04-9

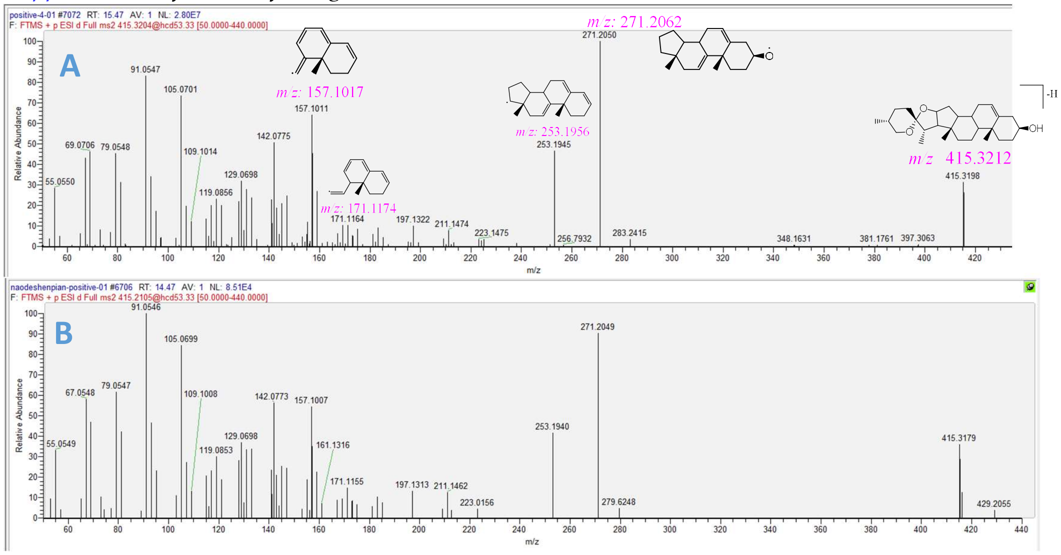

Fig. S66.1 The main results of standard diosgenin(CAS 512-04-9, $C_{27}H_{42}O_3$)and its corresponding peak in the TIC diagram using UPLC-Q-Orbitrap-MS analysis. (**A**) The MS/MS fragments of standard diosgenin. (**B**) The MS/MS spectra from chromatographic peak in the Naodeshengpian extract.

Note: The *m/z* values in purple are the calculated ones. The *m/z* calculation was based on the relative atomic masses of C (12.0000), H (1.007825), O (15.994915), and N (14.003074)[1].

Identification: As seen in Fig. S66.1, the R.T. value, molecular ion peak, MS/MS spectra, and characteristic pears were highly similar. Thus, the chromatographic peak in the Naodeshengpian extract was identified as diosgenin(CAS 512-04-9, $C_{27}H_{42}O_3$).

Suppl. 67 Identification of chloesteryl acetate CAS 604-35-3

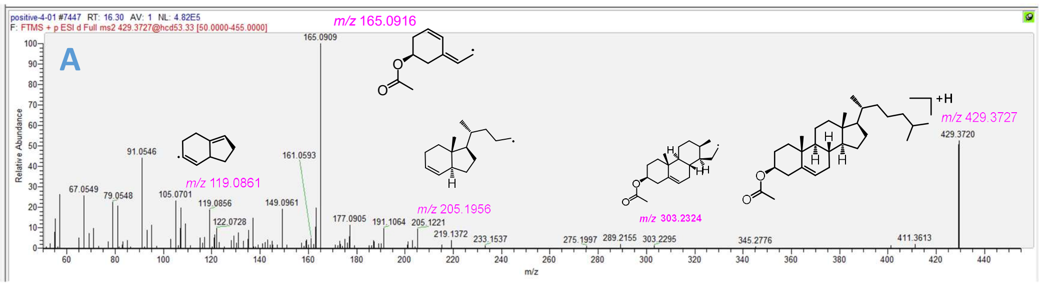

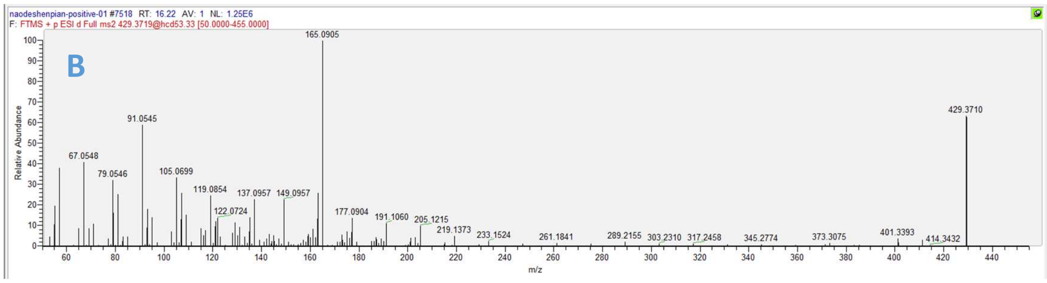

Fig. S67.1 The main results of standard chloesteryl acetate(CAS 604-35-3, $C_{29}H_{48}O_2$)and its corresponding peak in the TIC diagram using UPLC-Q-Orbitrap-MS analysis. (**A**) The MS/MS fragments of standard chloesteryl acetate. (**B**) The MS/MS spectra from chromatographic peak in the Naodeshengpian extract.

Note: The *m/z* values in purple are the calculated ones. The *m/z* calculation was based on the relative atomic masses of C (12.0000), H (1.007825), O (15.994915), and N (14.003074)[1].

Identification: As seen in Fig. S67.1, the R.T. value, molecular ion peak, MS/MS spectra, and characteristic pears were highly similar. Thus, the chromatographic peak in the Naodeshengpian extract was identified as chloesteryl acetate(CAS 604-35-3, $C_{29}H_{48}O_2$).

Suppl. 68 Identification of (+)-4-cholesten-3-one   CAS 601-57-0

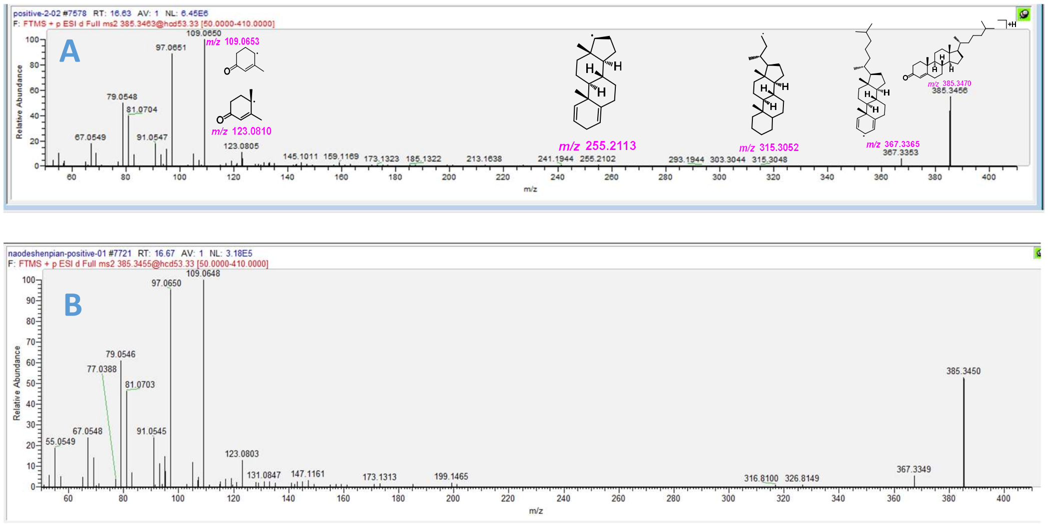

Fig. S68.1 The main results of standard (+)-4-cholesten-3-one(CAS 601-57-0, $C_{27}H_{44}O$)and its corresponding peak in the TIC diagram using UPLC-Q-Orbitrap-MS analysis. (**A**) The MS/MS fragments of standard (+)-4-cholesten-3-one. (**B**) The MS/MS spectra from chromatographic peak in the Naodeshengpian extract.

Note: The *m/z* values in purple are the calculated ones. The *m/z* calculation was based on the relative atomic masses of C (12.0000), H (1.007825), O (15.994915), and N (14.003074)[1].

Identification: As seen in Fig. S68.1, the R.T. value, molecular ion peak, MS/MS spectra, and characteristic pears were highly similar. Thus, the chromatographic peak in the Naodeshengpian extract was identified as (+)-4-cholesten-3-one(CAS 601-57-0, $C_{27}H_{44}O$).

Table s69. Relevant information on Semi-quantification of five Q-markers.

**Q-marker**	**Regression equation**	**Linear range μg**	**R**	**Area Mean ± SD (n=3)**	**Semi-quantification（%, Mean ± SD, n=3)**
puerarin（**12**）	y=29558661458.00x+28629582.52	0.003—0.09	0.9906	27797149904.83±4685068214.04	1.044±0.176
notoginsenoside R1（**36**）	y=1785224130.15x	0.000—0.09	0.9971	204957784.96±10284503.43	0.128±0.001
HSYA（**7**）	y=8198235744.27x-6664626.92	0.003—0.09	0.9994	279555302.82±17560673.84	0.039±0.002
levistilide A（**65**）	y=115338307380.79x-571258466.24	0.003—0.09	0.9992	6665965805.48±52682,021.52	0.070±0.006
citric acid（**2**）	y=19328804671.30x-84165248.88	0.003—0.09	0.9984	14240900956.59±359376499.50	0.822±0.021

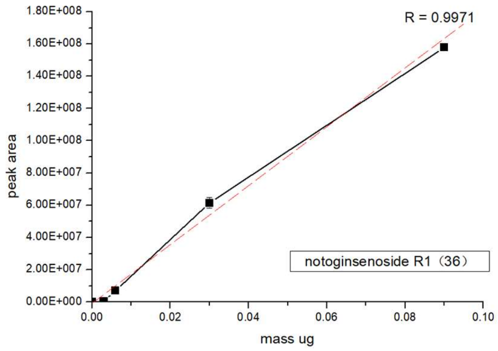

Fig. s69 A typical linear regression curve (taking notoginsenoside R1 as the example)

Note：Inject samples were at five concentrations, i.e., 0, 1.0, 2.0, 10.0, 30.0 μg/mL, which corresponds to 0.000, 0.003, 0.006, 0.03, and 0.09 μg. The injection volume was 3 μL.

Reference

[1] J.H., G., *Mass spectrometry*. 2013, Beijing: Science press.
